# Scoping review: potential harm from school‐based group mental health interventions

**DOI:** 10.1111/camh.12760

**Published:** 2025-03-18

**Authors:** Carolina Guzman‐Holst, Rowan Streckfuss Davis, Jack L. Andrews, Lucy Foulkes

**Affiliations:** ^1^ Department of Experimental Psychology University of Oxford Oxford UK

**Keywords:** School, interventions, harms, adolescents, CBT, mindfulness

## Abstract

**Background:**

A growing body of evidence demonstrates that school‐based mental health interventions may be potentially harmful. We define potential harm as any negative outcome or adverse event that could plausibly be linked to an intervention. In this scoping review, we examine three areas: the types of potential harms and adverse events reported in school‐based mental health interventions; the subgroups of children and adolescents at heightened risk; and the proposed explanations for these potential harms.

**Methods:**

We searched eight databases (1960–2023), performed an author search and hand‐searched for published and unpublished studies that evaluated controlled trials of school‐based group mental health interventions based on cognitive‐behavioural therapy and/or mindfulness techniques, with the aim of reducing or preventing internalising symptoms or increasing wellbeing. Two independent raters screened studies for eligibility and assessed study quality using Cochrane tools. From eligible studies, we reviewed those that reported at least one negative outcome.

**Results:**

Ten out of 112 (8.93%) interventions (described in 120 studies) reported at least one negative outcome such as a decrease in wellbeing or an increase in depression or anxiety. Three out of 112 interventions (2.68%) reported the occurrence of specific adverse events, none of which were linked to the intervention. Of the 15/120 studies rated as high quality (i.e. those with low risk of bias), 5/15 (33.33%) reported at least one negative outcome. Negative outcomes were found for a number of subgroups including individuals deemed at high risk of mental health problems, male participants, younger children and children eligible for free school meals. About half (54.5%) of the studies acknowledged that the content of the intervention itself might have led to the negative outcome.

**Conclusion:**

To design and implement effective school‐based mental health interventions, the issues of potential harm and their related measurement and reporting challenges must be addressed.

**Key Practitioner Message**

**What is known?**

- A number of recent studies have shown that school‐based mental health interventions based on principles from cognitive behavioural therapy (CBT) or mindfulness can lead to potential harm
- Despite the immense amount of research and public interest in school‐based interventions and young people's mental health more broadly, there has been limited exploration into why some interventions may lead to potential harm for some young people

**What is new?**

- This scoping review examines the existing evidence of potential harm from school‐based mental health interventions based on principles of CBT and/or mindfulness and explores who is most at risk and why these potential harms may occur

**What is significant for clinical practice?**

- We found that a minority of interventions reported at least one negative outcome, and when they did, it often occurred among specific subgroups of young people
- There are likely to be individual differences in how young people experience and respond to school‐based mental health interventions; therefore, teaching mental health lessons to all young people might not always be appropriate as itcould lead to potential harm

## Introduction

School‐based mental health interventions are designed to reduce or prevent mental health problems in young people. In universal interventions, information is delivered to whole classes of students, regardless of need, while targeted interventions, including both selective and indicated strategies, are delivered to smaller groups of students deemed to be at risk or who are already experiencing mental health problems (Gordon, 1983). Delivering such interventions to groups at school makes sense, since this is where young people spend the majority of their waking hours. Meta‐analyses to date indicate that school‐based mental health interventions can reduce symptoms of mental health problems in young people, although effect sizes vary considerably and tend to be larger for targeted, relative to universal, interventions (Fisak, Griffin, Nelson, Gallegos‐Guajardo, & Davila, 2023; Werner‐Seidler et al., 2021; Zhang, Wang, & Neitzel, 2023).

A number of recent studies have shown that school‐based mental health interventions based on principles from cognitive behavioural therapy (CBT) or mindfulness can lead to potential harm (Foulkes, Andrews, Reardon, & Stringaris, 2023; Foulkes & Stringaris, 2023). We define *potential harm* as any negative outcome or adverse event that could plausibly be linked to the school intervention itself (Foulkes et al., 2023). We use the word ‘potential’ to describe harm, as a negative outcome (e.g. decrease in wellbeing) might not always be considered harmful, especially if experienced mildly or transiently. For example, one universal CBT‐based intervention was found to increase internalising symptoms in the intervention group but not in the control group at six and 12 months post intervention (Andrews et al., 2022). Other studies have found negative outcomes for specific subgroups. For example, one CBT‐based intervention led to an increase in anxiety in the subgroup of children eligible for free school meals (an indicator of low parental income) and not in comparable children who received their usual classes (Wigelsworth et al., 2018). Another CBT‐based intervention led to an increase in depressive symptoms and feelings of personal failure in those who already had elevated mental health symptoms at baseline in the intervention group but not in the control group (Stallard et al., 2013). Similar results were found in a mindfulness‐based intervention, where participants at high risk of mental health problems at baseline in the intervention group reported increased symptoms of depression and decreased wellbeing compared to the control group (Montero‐Marin et al., 2022). Beyond mental health outcomes, a CBT‐based depression prevention programme found a decrease in prosocial behaviour in adolescents in the intervention group but no change in the control group that received their usual lessons (Seely, Gaskins, Pössel, & Hautzinger, 2023). It is possible that school‐based mental health interventions can also lead to specific adverse events, such as a self‐harm attempt, although the frequency and nature of these events are unclear, as many studies do not publish these details: one review of 12 universal school mental health interventions found that only one provided information in the final paper about recording adverse events (Mackenzie & Williams, 2018).

Qualitative studies offer further evidence that some young people have negative experiences in school‐based mental health interventions. In qualitative studies of school mindfulness programmes, while some young people describe the lessons as helpful and interesting, others find them distressing or confusing and say that the exercises made them focus on negative thoughts more, made them cry, or made them frustrated because they felt they could not do what was required (Bastounis, Callaghan, Lykomitrou, Aubeeluck, & Michail, 2017; Miller, Crane, Medlicott, Robson, & Taylor, 2023). Others said they did not understand why they were doing the lessons, or said the lessons limited their time for other activities such as practical problem‐solving, which they felt would have been more helpful for managing their feelings (Miller et al., 2023). Interview studies have found that being encouraged to think about negative thoughts in universal CBT‐based interventions made some young people feel low, even when they had initially felt positive (Garmy, Berg, & Clausson, 2015; Lindholm & Zetterqvist Nelson, 2015). Lastly, a large‐scale mixed‐method survey found that some young people considered a CBT‐based school mental health intervention unengaging or irrelevant to their own lives (Peters et al., 2023). Together, the quantitative and qualitative evidence to date indicate that there are likely considerable individual differences in how young people experience and respond to school‐based mental health interventions, and that for at least some individuals, these interventions could be potentially harmful.

Despite the immense amount of research and public interest in these interventions and young people's mental health more broadly, there has been limited exploration into why some interventions lead to potential harm for some young people. The current study is a scoping review that aims to map out the existing evidence of potential harm from school‐based mental health interventions focusing on principles drawn from CBT and/or mindfulness techniques, as to date these are the interventions that have found negative outcomes and are more widely used in school contexts. Our primary research questions are as follows:

1. What evidence is there that group school‐based mental health interventions that use CBT and/or mindfulness techniques can lead to potential harm?
2. Are there subgroups of young people who are particularly likely to experience potential harm from these interventions?

Our secondary research question:

3 What are the proposed explanations for why these potential harms occur?

## Methods

### Protocol and registration

This scoping review was pre‐registered with the Open Science Framework (OSF; https://osf.io/r9f4q) on 24 July 2023, following PRISMA guidelines for scoping reviews (Tricco et al., 2018). Deviations from our protocol are noted in Supplement S1.

### Information sources and search strategy

We searched databases for original research pertaining to school‐based mental health interventions: Education Collection (ProQuest); Embase (Ovid); ERIC (EBSCO); Global Thesis and Abstract (ProQuest); Medline (Ovid); PsycINFO (Ovid); PsyArXiv; and SCOPUS. Databases were selected to capture studies published in journals covering education, psychology and health‐related disciplines. To decrease publication bias, preprints, doctoral thesis and book chapters were included, in addition to journal articles. Searches were run for publications between 1 January 1960, as this is approximately when school mental health interventions began to emerge in the literature (Morse, Cutler, & Fink, 1964), and 16 December 2023, when we conducted our search. Additional studies were hand‐searched from protocols and cited studies in meta‐analyses. Author searches were conducted for authors that we were aware had published school‐based mental health trials with negative outcomes (Jack Andrews, Willem Kuyken, Hayley Seely, Paul Stallard and Michael Wigelsworth). To avoid repetition, we ran these author searches in one database only (PsychINFO). The search strategies were developed through consultation with an experienced subject librarian.

Our search terms are detailed below. Query strings were slightly adapted for each database (see Supplement S2, Table S1). Line 1 was searched in titles only, and lines 2–4 were searched in titles and abstracts. Lines 1 to 4 were combined using the Boolean operator ‘AND’.

1. intervention* OR program* OR trial OR train* OR curriculum
2. school*
3. mindfulness OR meditation OR cbt OR ‘cognitive behavio* therapy’
4. mental OR depress* OR mood OR anxi* OR internali?ing OR emotional problem* OR emotional functioning OR emotional symptom* OR emotional difficult* OR wellbeing OR well‐being

### Selection criteria

For a summary of selection criteria see Table 1. Studies were included if they evaluated outcomes of a mental health intervention delivered in a universal or targeted group format in schools to young people aged 4–19 years old. Interventions must have taught principles based on cognitive behavioural therapy (CBT), mindfulness or both, with the primary or secondary aim to reduce or prevent internalising symptoms (including measures of depressive and anxiety symptoms, mood and/or emotional problems) or increase wellbeing. To be included in our review, the study must have reported negative outcomes for at least one of its primary or secondary outcomes. ‘Negative outcomes’ refers to participants either experiencing an increase in an adverse construct (e.g. depressive symptoms) or a decrease in a desirable construct (e.g. wellbeing). Negative outcomes could include constructs beyond mental health symptoms (e.g. a decrease in prosocial behaviour). Separately, in addition to negative outcomes, we also examined studies that reported adverse events, defined as unfavourable medical occurrences (e.g. hospitalisation and suicide attempts; Foulkes, Andrews, Reardon, & Stringaris, 2024). While only studies with negative outcomes are included for review, we retained the total number of studies that met all other criteria (i.e. eligible studies) to obtain the proportion of studies reporting negative outcomes.

**Table 1:** Summary of inclusion and exclusion criteria

Criteria	Include	Exclude
Population and setting	Young people (aged 4–19) in schools	Young adults and adults (20+) Alternative education settings Online interventions with no taught component
Interventions	Mental health intervention delivered in a universal or targeted group format using principles based on CBT and/ or mindfulness with the goal or reducing or preventing internalising symptoms	Interventions using other modalities in addition to CBT and/or mindfulness Interventions adapted to address specific concern
Comparators	Passive or active control group that did not receive the intervention	Active control group receiving another mental health intervention
Outcomes	Negative outcomes' such as an increase in an adverse construct or a decrease in a desirable construct. Adverse events such as unfavourable medical occurrences	No negative outcomes or adverse events reported
Methods	Age‐appropriate, standardised and validated measures	Unstandardised, unvalidated measures Measures designed for adults
Other	For studies with multiple publications, the most relevant study (after assessing against all eligibility criteria) or studies presenting subgroup analyses	For studies with multiple publications, irrelevant studies or those with no subgroup details

CBT, cognitive behavioural therapy.

To be included in our review, the study design must have included at least one experimental group that received a CBT‐based and/or mindfulness‐based intervention and a control group that did not (i.e. we included waitlist, passive and teaching‐as‐usual control groups and any others that did not involve CBT, mindfulness or other mental health intervention additional to usual teaching). We included studies that evaluated outcomes using age‐appropriate, standardised and validated measures that displayed good internal consistency (α ≧ .6 in either the current study or existing literature) and previous evidence of construct validity. For studies describing interventions with multiple publications, the most relevant study was included after assessing them against all eligibility criteria. The only exception to this was if subgroup analyses were presented in a separate publication. For eligible articles presenting only subgroup analyses, we checked the corresponding primary study papers for recording of adverse events. Studies were excluded if the intervention was delivered in an alternative education setting (e.g. pupil referral unit or schools for those with special educational needs; see Supplement S2, Table S2), used CBT and/or mindfulness in combination with another modality (e.g. mindfulness with yoga), was adapted to address a specific concern (e.g. social anxiety and trauma) or delivered entirely online with no taught components (see Supplement S2, Table S3). For details of all excluded studies, see Supplement S2, Table S4.

### Study selection and screening

**Figure 1:**
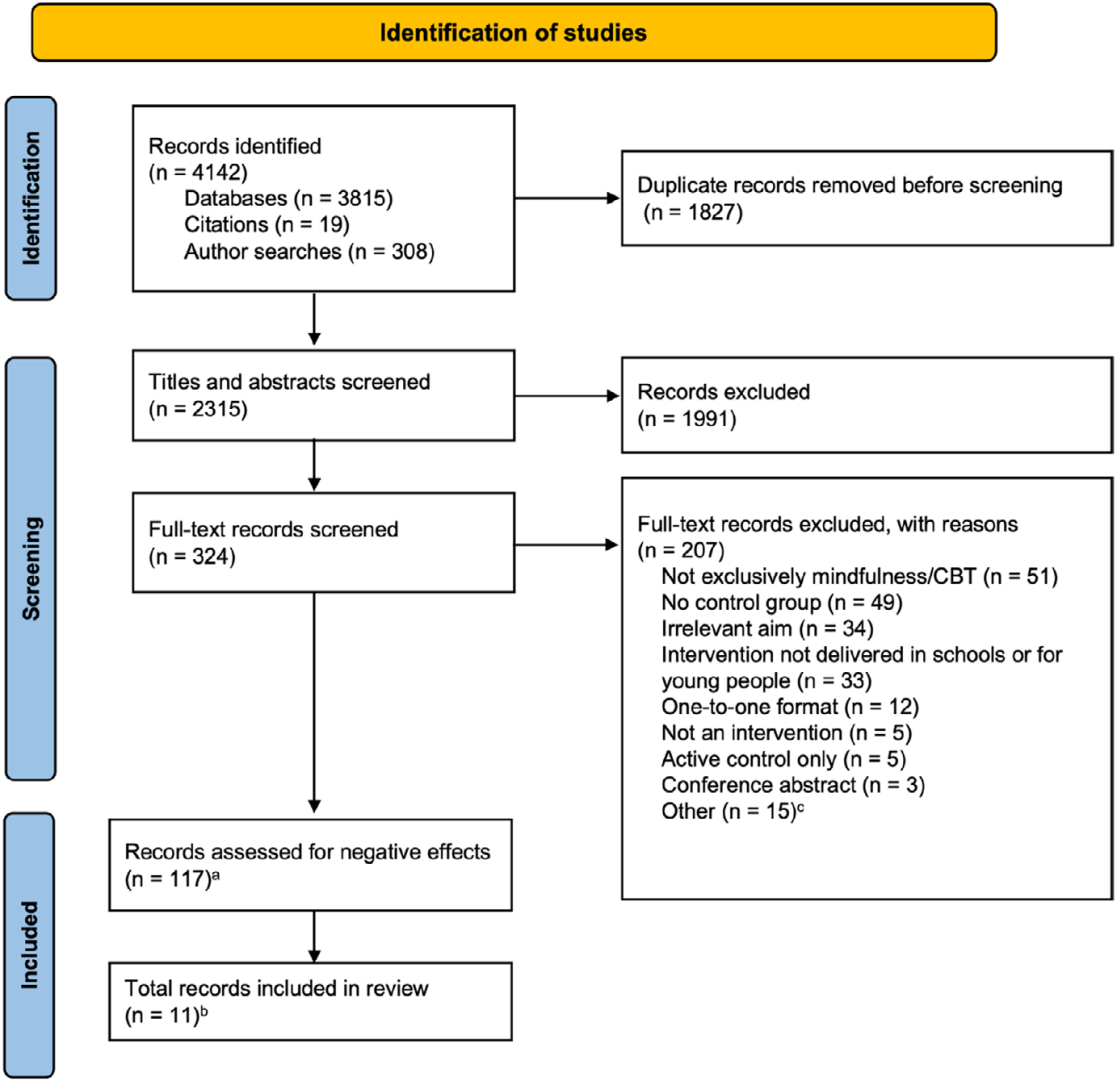
PRISMA study flowchart. See Supplement S2 for more details. ^a^117 records describe 120 studies (112 interventions). ^b^11 records describe 11 studies (10 interventions) with negative effects. ^c^The other category includes records with duplicate samples (*n* = 8), publications that could not be accessed (*n* = 4) publications not in English (*n* = 1), publications where the thesis was embargoed (*n* = 1), and publications with missing information (*n* = 1)

Figure 1 illustrates the study selection and screening process. One author (CGH) performed the initial search, returning 4142 records. After removing duplicates, two authors (RD and CGH) independently screened titles and abstracts for the resulting 2315 publications. In total, 324 records were eligible for full‐text screening and were reviewed by CGH, with a 10% check by RD, leaving 117 records (describing 120 studies and 112 interventions). Note that *record* refers to the total number of articles/reports included; *study* refers to the total number of individual studies across all records (sometimes results of multiple studies are published in the same record); and *intervention* refers to the total number of interventions reported across all studies (sometimes different analyses from the same intervention, with the same sample, are reported across multiple studies). These 117 records were eligible to be screened for negative outcomes and adverse events. In addition to our initial search, we conducted a secondary screening of the word ‘adverse’ in the main text of all eligible 117 records to identify other adverse events that might have been missed in our primary search. Inter‐rater reliability between RD and CGH for full‐text screening was calculated (*k* = .88) and classified as ‘substantial agreement’ (Landis & Koch,  1977). Any disagreements regarding eligibility were discussed and resolved between CGH and RD, with input from LF. For all study selection and screening processes, the systematic review software Rayyan was used (Ouzzani et al.,  2016). More details on study screening can be found in Supplement S2.

### Data extraction

We extracted the following study characteristics for studies reporting negative outcomes: study design, country, intervention name and type (i.e. universal or targeted delivery, CBT or mindfulness), sample size, participant age (range and mean), intervention duration and outcomes measured. For any negative outcome, the following information was extracted: type of negative outcome, effect sizes as calculated by each study author, and proposed explanations for the negative outcomes. For articles where adverse events were mentioned, we extracted the following additional information: article type (i.e. main study, follow‐up, subgroup analysis, thesis, or pilot study); whether adverse events were reported and, if so, the details of any event(s). RD completed data extraction for study characteristics and adverse events and CGH for negative outcomes.

### Assessment of methodological quality (risk of bias)

The Cochrane Collaboration's revised tool for risk of bias (Higgins et al., 2011) was used to assess the risk of bias and quality of included studies in five domains: bias arising from the randomisation process; bias due to deviations from intended interventions; bias due to missing outcome data; bias in measurement of the outcome; and bias in selection of the reported result. We used the tool specific for cluster randomised controlled studies (ROB2 – Cluster) and individually randomised controlled studies (ROB2; Higgins et al., 2011). CGH and JLA completed 50% of the risk of bias assessment each.

## Results

### Search results

A total of 2315 abstracts were reviewed. Of these, 324 records were included for full‐text review and 117 records (describing 120 studies and 112 interventions) were eligible for inclusion. Eleven records (describing 11 studies and 10 interventions) reported negative outcomes and thus make up our final selection of studies to review (see Figure 1).

### Study characteristics

Characteristics for 10 intervention trials reporting negative outcomes are presented in Table 2. Sample sizes ranged from 127 to 8376 students. Study participants' mean ages varied between 12 and 16 years of age. 60% (*n* = 6) of interventions were based on cognitive‐behavioural therapy (CBT) techniques and 40% (*n* = 4) were based on mindfulness techniques. All studies assessed universal interventions and came from high‐income countries: England (*n* = 3), Australia (*n* = 3), USA (*n* = 2), Canada (*n* = 1) and Germany (*n* = 1). Studies shared similar design characteristics; 90% (*n* = 9) of studies were cluster‐randomised and 10% (*n* = 1) were individually randomised. Almost all of the studies (90%; *n* = 9) had more than one measure of internalising symptoms or wellbeing. Interventions delivered anywhere between 6 and 12 sessions, which lasted between 30 min and 1 hr 30 min.

**Table 2:** Characteristics of studies that found negative outcomes

Authors	Study design	Modality	Country	Sample size at baseline	Participant age (M)	Outcomes	Risk of bias^a^
Andrews et al. (2022)	CRCT	CBT	Australia	3200	12–14 (13.6)	^P^ Internalising problems (SDQ) ^S^ Depression (PHQ‐8) ^S^ Anxiety (GAD‐7)	Low risk
Frank et al. (2021)	CRCT	Mindfulness	USA	251	16–17 (16.0)	^P^ Mindfulness (CAMM) ^P^ Self‐compassion (SCS‐SF) ^P^ Emotion regulation (DERS) ^P^ Depression (PHQ‐8) ^P^ Anxiety (GAD‐7) ^P^ Rumination (RRQ) ^P^ Stress (ASQ) ^P^ Somatisation (CSI) ^P^ Sleep (ASWS) ^P^ Social connectedness (SCC‐R) ^P^ Mind‐wandering (MWQ) ^P^ Growth mindset (IT) ^P^ Substance use (SII) ^P^ Negative substance use consequences (YAAPST)	Low risk
Johnson, Burke, Brinkman, and Wade (2016)	CRCT	Mindfulness	Australia	308	12–14 (13.6)	^P^ Anxiety and depression (DASS‐21) ^P^ Weight/shape concern (EDE‐Q) ^P^ Wellbeing (WEMWBS) ^S^ Mindfulness (CAMM) ^S^ Emotional dysregulation (DERS) ^S^ Self‐compassion (SCS)	Some concerns
Johnson and Wade (2021)	CRCT	Mindfulness	Australia	434	13–14 (13.7) and 15–16 (15.5)	^P^ Mindfulness (CHIME‐A) ^P^ Depression (DASS‐21) ^P^ Anxiety (GAD‐7) ^P^ Weight/shape concern (EDE‐Q) ^P^ Wellbeing (WEMWBS)	Some concerns
Klim‐Conforti et al. (2021)	RCT	CBT	Canada	603	11–14 (N/A)	^P^ Suicidality (LPI) ^S^ Life problems (LPI) ^S^ Anxiety and depression (RCADS)	High risk
Kuyken et al. (2022) (subgroup analysis: Montero‐Marin et al., 2022)	CRCT	Mindfulness	UK	8376	11–14 (12.6)	^P^ Risk for depression (CES‐D) ^P^ Social–emotional and behavioural functioning (SDQ) ^P^ Well‐being (WEMWBS) ^S^ Executive function (BRIEF‐2) ^S^ Anxiety (RCADS) ^S^ Social–emotional and behavioural functioning (TSDQ) ^S^ School climate (SCCS) ^S^ Mindfulness skills (CAMM)	Low risk
Seely et al. (2023)	CRCT	CBT	Germany	646	13–14 (14.0)	^P^ Social–emotional and behavioural functioning (SDQ)	Some concerns
Stallard et al. (2012)	CRCT	CBT	UK	5030	12–16 (14.0)	^P^ Depressive symptoms (SMFQ) ^S^ Personal failure (CATS) ^S^ Self‐worth and acceptance (RSES) ^S^ Anxiety and depressive symptoms (RCADS) ^S^ Bullying (OBVQ)	Low risk
Stoppelbein (2003)	CRCT	CBT	USA	127	15–16 (15.0)	^P^ Depressive symptoms (CDI) ^P^ Dysfunctional attitudes (DAS) ^P^ Cognitive style (CTI‐C) ^P^ Attributional style (CASQ)	High risk
Wigelsworth et al. (2018)	CRCT	CBT	UK	3284	9–10 (N/A)	^P^ Academic attainment ^P^ Worry (PSWQ‐C) ^S^ Anxiety and depressive symptoms (RCADS 25) ^S^ Socio‐emotional and behavioural functioning (SDQ)	Some concerns

^P^ = Primary outcome; ^S^ = Secondary outcome. ASQ, Adolescent Stress Questionnaire; ASWS, Adolescent Sleep–Wake Scale; BRIEF‐2, Behaviour Rating Inventory of Executive Function; CBT, cognitive behavioural therapy; CAMM, Child and Adolescent Mindfulness Measure; CASQ, Children's Attributional Style Questionnaire; CATS=Children's Automatic Thoughts Scale; CDI=Children's Depression Inventory; CES–D=Center for Epidemiological Studies Depression scale; CHIME‐A, Comprehensive Inventory of Mindfulness Experiences—Adolescents; CSI, Children's Somatization Inventory; CTI‐C, Cognitive triad inventory‐Child version; DAS , Dysfunctional Attitudes Scale; DASS‐2, Depression Anxiety Stress Scale; DERS, Difficulties in Emotion Regulation Scale; EDE‐Q, Eating Disorder Examination–Questionnaire; GAD‐7, Generalised Anxiety Disorder seven item scale; IT, Implicit Theories of Intelligence Scale for Children; LPI, Life Problems Inventory; MWQ, Mind Wandering Questionnaire; OBVQ, Olweus Bully/Victim Questionnaire; PHQ‐8, Patient Health Questionnaire–8; RCADS, Revised Child Anxiety and Depression Scale; RRQ, Rumination and Reflection Questionnaire; RSES, Rosenberg Self‐esteem Scale; SCC‐R, Social Connectedness Scale; SCCS, School Climate and Connectedness Survey; SCS, Self‐compassion scale; SDQ, Strengths and Difficulties Questionnaire; SII=Substance Initiation Index; SMFQ, Short Mood and Feelings Questionnaire; WEMWBS, Youth Self‐Report Warwick‐Edinburgh Mental Well‐being Scale; YAAPST, Young Adult Alcohol Problems Screening Test.

^a^ Risk of bias was assessed using the Cochrane Collaboration tool.

### Quality assessment and risk of bias in included studies

From the 11 included studies, five studies (45.5%) were rated as having a low risk of bias, four studies (36.4%) were rated as having some concerns for risk of bias, and two studies (18.2%) were rated as having a high risk of bias. A detailed quality assessment is shown in Supplement S3, Table S5 and Figure S1. All 120 eligible studies were reviewed for full‐text screening (note that one record, a thesis, described three studies and another record, an RCT, described two studies). In total, 33.33% (5/15) of studies with low risk of bias reported negative outcomes, as did 13.33% (4/30) of studies with some risk and 2.67% (2/75) of studies with a high risk of bias.

### Research question 1: What evidence is there that group school‐based mental health interventions that use CBT and/or mindfulness techniques can lead to potential harm?

Ten out of 112 eligible interventions (8.93%) reported negative outcomes in at least one outcome measure. Negative outcomes were reported in both primary and secondary outcomes, across a range of measures including wellbeing, depression, anxiety, general internalizing symptoms, hyperactivity/inattention difficulties, negative cognitions related to depression (e.g. thoughts of personal failure and dysfunctional attitudes), prosocial behaviour, mindfulness and impulsivity. Some of these effects were only found for specific subgroups (e.g. males, those eligible for free school meals and those with higher baseline mental health problems) while others were reported across all study participants. Details of the negative outcomes found in each study are described below, with a brief summary of the other findings from the study to provide context. Further details about each study can be found in Tables 2 and 3. Additional details on other outcomes reported in each study and intervention characteristics can be found in Supplement S4, Table S6.

**Table 3:** Description of negative outcomes found in included studies

Authors	Negative outcomes	Type	Description of effects and effect sizes^d^
Andrews et al. (2022)	Increase in internalising symptoms at 6‐months post and 12‐month follow‐up	Main analysis (primary outcome)	Adolescents in the intervention group reported a significant increase in internalising symptoms (SDQ) at 6‐months (*d* = 0.11) and at 12‐months (*d* = 0.06) compared to adolescents in the control group.
Frank et al. (2021)	Increase in difficulties engaging in goal directed behaviour at post‐ intervention	Main analysis (secondary outcome)	Adolescents in the intervention group reported a significant increase in difficulties engaging in goal directed behaviour (DERS) at post‐intervention (*d* = 0.37) compared to adolescents in the control group.
Johnson et al. (2016)	Increase in anxiety at the 3‐month follow‐up	Subgroup analysis (baseline characteristics)	Adolescents in the intervention group with low baseline levels of weight/shape concerns (*d* = 0.30, EDE‐Q), low baseline levels of depression (*d* = 0.27, DASS‐21) and males (*d* = 0.22) reported an increase in anxiety at the 3‐month follow‐up compared to equivalent adolescents in the control group.
Johnson and Wade (2021)	Decrease in aspects of mindfulness and wellbeing at 3‐month follow‐up and 9‐month follow‐up and weight/shape concerns at 9‐month follow‐up	Main analysis (primary outcome) Subgroup analysis (year group/age) Subgroup analysis (extra follow‐up^a^)	Main analysis (full sample): students in the intervention group scored lower in mindfulness decentering and non‐reactivity at the 3‐month follow‐up (*d* = −0.20, CHIME‐A) compared to students in the control group. Subgroup (year group/age): 13–14 year olds (compared to 15–16 year olds) in the intervention group scored lower in wellbeing (*d* = −0.25, WEMWBS) and two aspects of mindfulness: awareness of external environment (*d* = −0.30, CHIME‐A) and decentering and nonreactivity (*d* = −0.39, CHIME‐A) at the 3‐month follow‐up compared to controls. Subgroup (participating in extra follow‐up): students in the intervention group scored lower in wellbeing (*d* = −0.41, CHIME‐A) and mindfulness decentering and nonreactivity (*d* = −0.43, CHIME‐A) at the 3‐month follow up and wellbeing (*d* = −0.40, WEMWBS) and weight/shape concerns (*d* = −0.35, EDE‐Q) at 9‐month follow‐up compared to controls
Klim‐Conforti et al. (2021)	Increase in impulsivity at post‐intervention	Subgroup analysis (gender)	Males in the intervention group had higher impulsivity scores (LPI) at post‐intervention (*t* = −2.52, *df* = 97, *p* = 01) compared to males in the control group who reported a decrease in impulsivity.^b^
Kuyken et al. (2022)	Increase in hyperactivity/inattention symptoms, panic disorder symptoms, obsessive‐compulsive disorder symptoms, total anxiety symptoms, emotional problems and a decrease in mindfulness skills	Main analysis (primary and secondary outcomes)	The intervention group reported an increase in hyperactivity/inattention symptoms (SDQ) at postintervention (AMD [95% CI] = 0.015 [0.04, 0.3]; *p* = .01) and 12‐month follow‐up (AMD [95% CI] = 0.015 [0.04, 0.3]; *p* = .01), an increase in panic disorder symptoms (RCADS) at post (AMD [95% CI] = 0.019 [0.03, 0.9]; *p* = .04), an increase in obsessive‐compulsive disorder symptoms (RCADS) at post (AMD [95% CI] = 0.016 [0.01, 0.5]; *p* = .04), an increase in total anxiety symptoms (RCADS) at post (AMD [95% CI] = 1.3 [−0.2 to 2.7]; *p* = .08) and teacher‐reported emotional symptoms (TSDQ) at follow‐up (AMD [95% CI] = 0.053 [0.1, 0.5]; *p* = .01). The intervention group also reported a decrease in mindfulness skills (CAMM) at post (AMD [95% CI] = 0.021 [−1.2, −0.01]; *p* = .04) compared to the control group.
Montero‐Marin et al. (2022) (subgroup analysis for Kuyken et al., 2022)	Increase in depressive symptoms and decrease in wellbeing at post‐intervention and 12‐month follow‐up	Subgroup analysis (individuals at high risk of mental health problems) Subgroup analysis (age)	High‐risk adolescents in the intervention group reported an increase in depressive symptoms (CES‐D) at post‐intervention (AMD [95% CI] = 1.40 [0.27, 2.53]) and 1‐year follow up (AMD [95% CI] = 1.47 [0.37, 2.57]); decrease in wellbeing (WEMWBS) at post‐intervention (AMD [95% CI] = −1.10 [−1.98, −0.22]) and 12‐month follow up (AMD [95% CI] = −0.88 [−1.71, −0.05]) compared to equivalent individuals in the control group.
Seely et al. (2023)	Decrease in prosocial behaviour at post‐intervention	Primary analysis (primary outcome)	Adolescents in the intervention group reported a significant decrease in prosocial behaviour (*d* = 0.41, SDQ) at post‐intervention compared to adolescents in the control group. This effect was stronger for the MHP prevention group (delivered by a mental health professional).
Stallard et al. (2012)	Increase in depressive symptoms and automatic thoughts (i.e. feelings of personal failure) at 12‐month follow‐up	Primary analysis (primary and secondary outcomes) on sub‐population of individuals with high‐risk of mental health	Adolescents in the intervention group reported an increase in depressive symptoms (SMFQ) at 12‐months (AMD [95% CI] = 0.64 [0.06 to 1.21]) and an increase in automatic thoughts or thoughts of personal failure (CATS) at 12‐moths (AMD [95% CI] = 1.95 [0.25 to 3.66]) compared to adolescents in the control group. Note: effects were only found in analysis accounting for variables that were imbalanced at baseline
Stoppelbein (2003)	Increase in dysfunctional attitudes (i.e. cognitions related to depression) at post‐intervention	Subgroup analysis (subclinical depressive scores at baseline)	Adolescents with baseline subclinical depressive scores (i.e. higher levels of depression) in the intervention group reported a significant increase in dysfunctional attitudes (DAS) at post‐intervention (*d* = 0.50) compared to equivalent adolescents in the control group^c^.
Wigelsworth et al. (2018)	Increase in depression and anxiety at post‐intervention	Subgroup analysis (eligibility for free school meals)	Children eligible for free school meals in the intervention group reported a significant increase in depression and anxiety symptoms (RCADS) at post intervention (*g* = 0.25) compared to equivalent children in the control group.

AMD, adjusted mean difference; CI, confidence intervals; *d*, Cohen's *d*; *g*, Hedges *g*.

^a^ Students that participated in the first two rounds of intervention assessments in addition to a 9‐month follow‐up.

^b^ The authors of this study report a Welch's *t*‐test which cannot be used to compute effect sizes.

^c^ This study does not provide an effect size; therefore, the authors calculated Cohen's *d* with the unstandardised coefficient (*B* = 14.94), standard error (*SE* = 5.80) and standard deviation (*SD* = 30.74) resulting in *d* = 0.50, CI = 0.12–0.87.

^d^ See Table 1 for measurement acronyms.

Three out of 112 interventions (2.68%) reported adverse events, none of which were related to the intervention. The adverse events reported included one terminal illness diagnosis and one death, both in teachers in the intervention condition (Kuyken et al., 2022); indication of suicidal ideation in six participants, one in the control and five across the three intervention conditions (Poppelaars et al., 2016); and indication of suicidal ideation or self‐harm in five participants, all in the control condition (Britton et al., 2014).

### Research question 2: Are there subgroups of children/adolescents who are particularly likely to experience potential harm from these interventions?

Our findings show that in 55% (*n* = 6) of the studies identified as having negative outcomes, two studies found negative outcomes in both the main analysis and subgroup analysis, and four studies found negative outcomes only in the subgroup analysis (see Table 3). These studies found that subgroups of participants in the intervention conditions were more likely to experience negative outcomes than comparable participants in the control conditions. These included young people deemed at high risk of mental health problems (defined as those with higher levels of socio‐emotional‐behavioural difficulties, higher scores on risk of depression and lower wellbeing; Montero‐Marin et al., 2022), adolescents with subclinical levels of depression (those with higher levels of depression; Stoppelbein, 2003), male participants (Klim‐Conforti et al., 2021), younger children (Johnson & Wade, 2021), and children eligible for free‐school meals (Wigelsworth et al., 2018). In one study, subgroup analysis revealed that young people in the intervention group with low baseline levels of weight/shape concerns, depression or who identify as males reported an increase in anxiety outcomes at a follow‐up assessment (Johnson et al., 2016). The other 45% of studies not mentioned here do not report subgroup analyses (*n* = 4) or based their primary analysis on a high‐risk sample (*n* = 1).

### Research question 3: What are the proposed explanations for why these potential harms occur?

**Figure 2:**
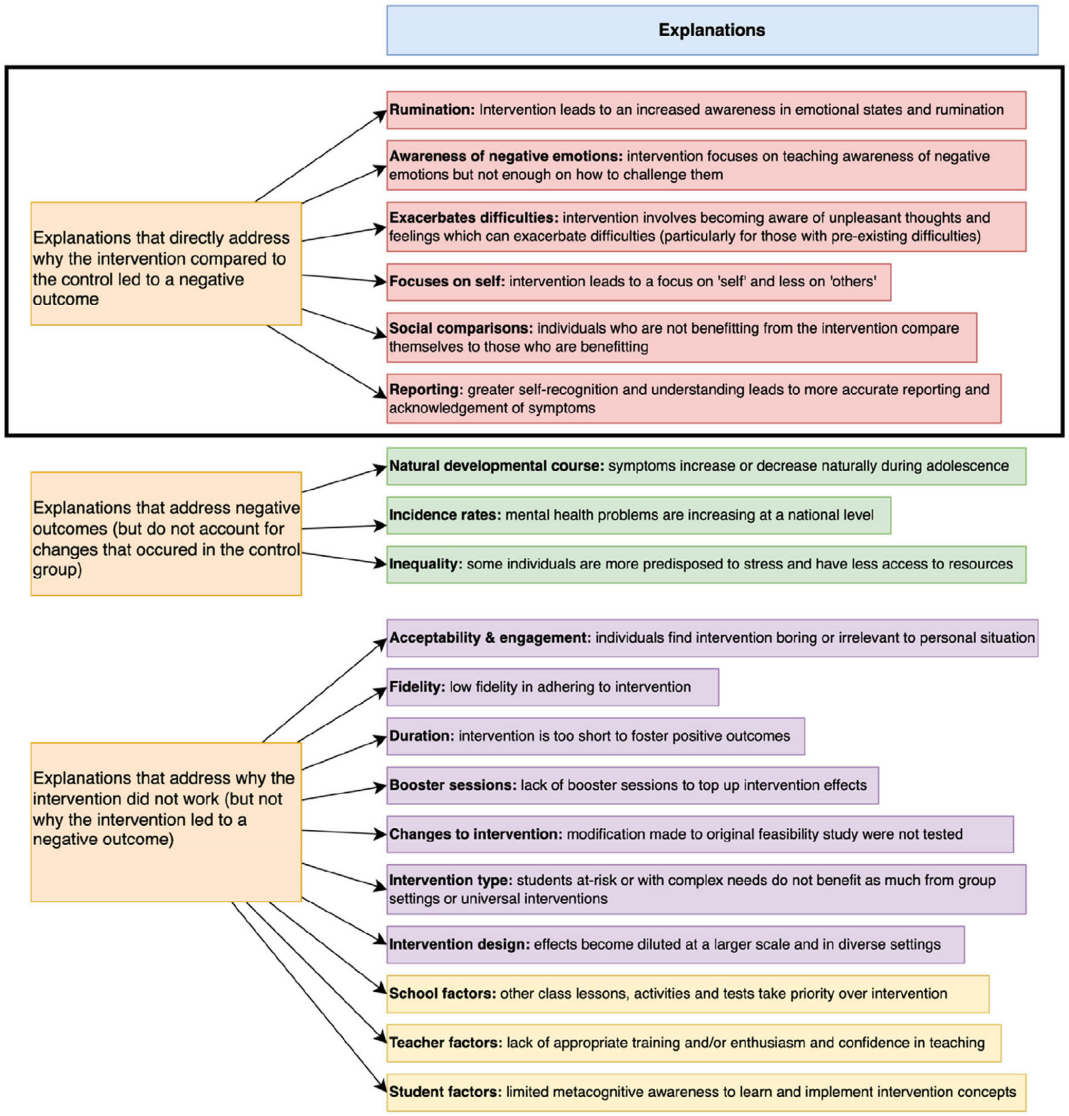
Proposed explanations for negative outcomes. The black box highlights the studies of particular interest, which provide direct explanations for negative outcomes

In the 10 interventions (11 studies) described above (see Supplement S4, Table S6 for specific intervention characteristics), two did not propose explanations for the negative outcome found (Frank et al., 2021; Klim‐Conforti et al., 2021). The others proposed several explanations for their negative outcomes, which are detailed here and summarised in Figure 2.

First, some authors suggested that negative outcomes reflected a natural developmental course in the construct, that is, that the scores would have decreased or increased anyway, regardless of intervention, because of developmental changes that occur during adolescence. Specifically, Andrews and colleagues suggested that the significant increase in internalising symptoms in the intervention group, as well as the control group, was consistent with rising incidence rates of depression and emotional problems in the general population (Andrews et al., 2022). However, while this explanation explains the main effect of time for both groups at the 18‐month follow‐up, it does not address the differences between the intervention and control groups at 6 and 12 months. Similarly, Seely and colleagues suggested that the decrease in prosocial behaviour seen in the CBT intervention group was because prosocial behaviour tends to decline in adolescence; however, this does not explain why this decline was not also seen in the control group (Seely et al., 2023). Relatedly, Wiglesworth and colleagues suggested that the increase in depression and anxiety found in the subgroup of children eligible for free school meals (in the CBT intervention group only) was likely linked to the fact that these children have less access to community resources and more exposure to stress, but this does not explain why such an increase was not found in the equivalent group of children in the control group (Wigelsworth et al., 2018).

Other explanations focused on the idea that having a school‐based mental health intervention increases young people's understanding of a specific difficulty and that a negative outcome therefore represents more accurate reporting rather than an actual deterioration in the construct. For example, Seely and colleagues suggested that there was a reduction in prosocial behaviour in the intervention group because these participants acquired knowledge of what it meant to be prosocial and gained increased awareness of their true (lower) level of prosocial behaviour; in contrast, participants in the control condition were not exposed to definitions of prosocial awareness and thus responded to the questionnaires at both time points with more basic knowledge and understanding of this construct (Seely et al., 2023). Similarly, Stallard and colleagues proposed that the increase in depressive symptoms and thoughts of personal failure in the intervention group may reflect greater self‐recognition and acknowledgment of existing symptoms; they suggest this did not occur in the control group because they did not learn about depression (Stallard et al., 2012). Lastly, Johnson and colleagues proposed that the negative outcomes found in males, those with low levels of baseline depression or those with weight/shape concerns could be due to increased awareness of emotional states as mindfulness increases (Johnson et al., 2016). In this study, males showed higher baseline scores for mindfulness, and the authors suggest that this may have amplified the effect of increased awareness of emotional states, leading males to report higher levels of anxiety post‐intervention (Johnson et al., 2016). While Johnson and colleagues acknowledged that they cannot explain the increases in anxiety for individuals with low levels of depression or weight/shape concerns at baseline, they note that one possibility is that the intervention increased their (previously low) awareness of emotional states, which may have become somewhat ruminative in nature.

Some studies offered explanations for why the intervention did not work (i.e. did not lead to positive outcomes). While this information is particularly relevant given that most of the studies found null effects in their primary outcomes, they do not address why the intervention may have had a negative outcome specifically. For example, Stallard and colleagues suggested that school‐level factors (such as compatibility with organisational objectives and school priorities) may have limited the effectiveness of the interventions (Stallard et al., 2012). They also suggested that programme‐specific factors (such as whether the content was interesting to participants or led by enthusiastic and confident facilitators) might have limited effectiveness, that potential positive effects may become diluted when implemented at a larger scale and under more diverse conditions, or that CBT skills taught in group settings may be insufficient to support individuals who have greater needs or higher levels of symptoms (Stallard et al., 2012). Lastly, they suggested that participants themselves may limit the effectiveness of the intervention because they do not perceive themselves as having symptoms and thus do not engage with the programme because they do not recognize its relevance to them (Stallard et al., 2012).

Authors evaluating the MYRIAD trial offered similar suggestions for why the mindfulness intervention did not lead to positive outcomes: that acceptability was mixed and therefore some participants did not engage with the intervention, and that the school teachers who delivered the intervention may not have had sufficient time or training to foster positive outcomes or maintain fidelity (Kuyken et al., 2022; Montero‐Marin et al., 2022). These authors also suggested that participants may have been too young to have the level of metacognitive awareness required to learn and implement mindfulness techniques (Kuyken et al., 2022; Montero‐Marin et al., 2022). Relatedly, Stoppelbein suggested that the programme was not successful because it was relatively brief and did not include booster sessions top‐ups (Stoppelbein, 2003), while Johnson and Wade suggested that any null effects might be due to modifications made from the original feasibility study (Johnson & Wade, 2021).

Some authors acknowledged that the content of the intervention itself may have led to the negative outcomes. For example, Seely and colleagues reported that the CBT‐based intervention may have had an iatrogenic effect and suggested two possible mechanisms: that this intervention may have led participants who were not benefiting from the intervention to compare themselves with others benefiting from the intervention in a negative manner, which might then lead to less empathy and less prosocial behaviour, or that the focus on one's own cognition and behaviours may have meant less focus on behaviour towards others (Seely et al., 2023). Stoppelbein suggested that an increase in negative cognitions, such as dysfunctional attitudes related to depression, may be a function of the programme itself, since it is focused on teaching participants to be aware of negative cognitions without teaching them to challenge these beliefs until the last few sessions (Stoppelbein, 2003). Similarly, MYRIAD authors noted that mindfulness training involves encouraging people to become more aware of their thoughts and feelings, including unpleasant ones, which may have exacerbated hyperactivity/inattention difficulties, emotional symptoms, and anxiety symptoms (Kuyken et al., 2022), particularly for individuals that had pre‐existing risks for mental health problems (Montero‐Marin et al., 2022). Stallard and colleagues acknowledged that the intervention itself may be potentially harmful, as it had detrimental effects on depressive symptoms and thoughts about personal failure, and while they do not speculate on potential mechanisms for these effects, they emphasise the need for future research in this area (Stallard et al., 2012). Both the MYRIAD authors and Wiglesworth and colleagues highlighted that individual differences are likely to be important, in that some subgroups seem to experience negative outcomes from interventions, but these may be concealed or cancelled out by positive effects when effects are averaged across the entire sample (Kuyken et al., 2022; Montero‐Marin et al., 2022; Wigelsworth et al., 2018).

## Discussion

This study reviewed over 30 years of research to assess the nature of potential harm from school‐based group mental health interventions that focus on CBT and/or mindfulness techniques and aim to reduce internalising symptoms or increase wellbeing. We define potential harm as any negative outcome or adverse event that could plausibly be linked to the intervention itself and assessed how frequently these occurred, whether they were more likely to occur in particular subgroups of young people, and what explanations (if any) were proposed for why these potential harms occur. We found that a minority of interventions (8.93%) among those eligible reported at least one negative outcome, and when they did, it often occurred among specific subgroups of young people. Among studies rated as high quality (low risk of bias), 33% reported at least one negative outcome. 45.5% of studies did not suggest direct explanations as to why these harms occurred only in the intervention group. Most papers did not report details of adverse events, but for those that did, they were not linked to the intervention. Together, the findings indicate that potential harm from school‐based mental health interventions is relatively infrequent but deserves careful attention in future intervention research, particularly regarding more vulnerable subgroups.

The negative outcomes found in the current review covered a range of constructs including wellbeing, internalising symptoms (including depression and anxiety), hyperactivity/inattention difficulties, negative cognitions related to depression, prosocial behaviour, mindfulness, and impulsivity. This review has demonstrated that negative outcomes are found only in a minority of school‐based CBT and/or mindfulness mental health interventions, which should offer some reassurance to the field. However, it is important to highlight the heterogeneity in the findings and to temper any undue optimism about the effectiveness of these interventions. Specifically, it is common to find null effects (Caldwell et al., 2019): for 50% of our included studies, there are no positive effects reported at all. Null and negative outcomes were especially likely to be reported in high‐quality trials with random allocation to conditions, larger sample sizes, and robust methodology (for example, those that adjust for baseline variables and clustering, correct for multiple comparisons, and conduct sensitivity analyses on missing data), perhaps because they are more confident in reporting and explaining all effects, including null and negative ones. There were also commonalities in intervention characteristics among studies reporting negative outcomes; all interventions were universal (instead of targeted), structured (e.g. following manuals or protocols), and reported adequate fidelity. However, there were also several differences, such as who delivered the intervention (e.g. teachers, external officers and researchers), the duration of programmes, and what materials they used (e.g. lessons, homework, practical activities). Further investigating commonalities in interventions' characteristics among studies reporting negative effects and those not reporting negative effects should be a matter of priority, as these may reveal potential sources of iatrogenic effects.

An important finding from the current review is that negative outcomes often occurred in subgroups. Specifically, 55% of our included studies found negative outcomes in subgroup analyses. These showed that young people deemed at high risk of mental health problems, male participants, younger children, children experiencing deprivation, and children experiencing fewer weight/shape concerns or depression reported worsening of mental health problems and/or wellbeing following an intervention. These findings demonstrate that there are likely to be considerable individual differences in how young people respond to school mental health interventions and that average effects (whether positive, negative or null) are unlikely to give the full picture about the impact of an intervention. Moreover, our results suggest there might be heterogeneity in iatrogenic effects even among similar subgroups (e.g. Montero‐Marin and colleagues found negative outcomes among subgroups experiencing higher level of symptoms at baseline, whereas Johnson and colleagues found negative outcomes among subgroups experiencing lower levels of symptoms at baseline). It is therefore essential that future intervention studies are powered to examine subgroup effects and plan for this analysis in their protocols (Foulkes et al., 2024).

It may be that, ultimately, these subgroup effects and individual differences demonstrate that universal interventions are not the right approach to preventing or reducing mental health problems in young people. Instead, a better approach in schools might be to assess individuals on baseline characteristics and personal preferences, and then triage them into different targeted interventions; this triaging system may involve a group of young people who require no intervention at all beyond what is typically delivered as usual practice. Alternatively, young people could be triaged into different versions of the same universal interventions (e.g. varying both in content and duration), so that individuals who might need more help receive a more intensive approach. It is important that schools or those leading the intervention involve young people in the co‐production of the intervention and/or empower young people to choose what version or dose of an intervention they want to engage with by presenting information about the intervention beforehand, and thus giving them the power and autonomy to decide their level of engagement with mental health content. This idea falls in line with a ‘proportionate universalism’ approach to public health, which suggests that interventions should be universally delivered but with an intensity and at a scale that is proportional to the population's level of need (Francis‐Oliviero, Cambon, Wittwer, Marmot, & Alla, 2020; Marmot, 2018). Indeed, it is possible that interventions might work best when delivered either at a higher ‘dosage’ or not at all, rather than at a mild dosage for everyone. To date, we are not aware of any research assessing this triaged approach in the context of school mental health interventions, and we note that there are many complexities and challenges with implementing proportionate universalism approaches, including identifying needs, applying proportionality, and evaluating and interpreting intervention results (Francis‐Oliviero et al., 2020).

In considering alternatives to universal approaches in schools, a range of targeted interventions can be explored. These might include indicated components such as one‐to‐one counselling sessions for personalised mental health support and group interventions (for example based on CBT or mindfulness) for individuals identified as at risk of mental health problems, providing a more focused therapeutic approach (e.g. Smith et al., 2015; van Starrenburg, Kuijpers, Kleinjan, Hutschemaekers, & Engels, 2017). Whole‐school initiatives to improve the overall school climate and culture or promote healthy behaviours can also foster a supportive environment for all students (Bonell et al., 2019; Hinze et al., 2024). Additionally, mental health interventions for school staff can also enhance the support network within the school (Roeser et al., 2022). Each approach has its advantages and drawbacks; for instance, targeted interventions may face issues of stigma and limited reach (Gronholm, Nye, & Michelson, 2018), while whole‐school approaches and peer‐led initiatives can be more scalable and accessible but might require significant time and resources to implement effectively. For all of these approaches, it is important to acknowledge that they may still cause potential harm to some individuals, particularly if they are not implemented correctly and following evidence‐based practices; potential harm should thus always be considered in future research.

In this review, we also examined whether school‐based mental health interventions reported adverse events. In the current review, we found that three out of the 112 interventions reported adverse events occurring, all of which found that they were not due to the intervention itself. It is likely that the other 109 interventions will have reported possible adverse events to their research team and ethics boards, as this is an ethical requirement but did not report this detail in the final publications (possibly because no such adverse events were found). We suggest that reporting details on adverse events in all post‐trial published outputs, even if none or few were found, is an important aspect of documenting the safety of school‐based mental health interventions (Foulkes et al., 2024). We recommend that possible adverse events are defined in the initial planning stages of the trial and that consideration is made regarding when adverse events might occur (for example, during data collection and homework sessions as well as the intervention itself) and who will have responsibility for recording this information and training relevant parties in this as part of a standard operating procedure (Foulkes et al., 2024).

Our secondary research question was to examine the proposed explanations offered by authors with regard to any identified negative outcomes with the intention that this might highlight key factors that can be investigated as moderators/mediators in future research. We found that five studies (five interventions) did not provide direct explanations as to why negative outcomes only occurred in the intervention group, while six studies (five interventions) provided some explanation. Most studies propose that inequality, school characteristics, barriers to implementation, individual‐level factors, and developmental change all contribute to determining whether an intervention is effective or not, rather than focusing on intervention‐specific negative outcomes (Johnson & Wade, 2021; Kuyken et al., 2022; Stallard et al., 2012; Stoppelbein, 2003). This indicates that, as a field, we have limited evidence or theory about the potential mechanisms that underlie negative outcomes in school‐based mental health interventions. It remains unclear whether these effects represent a genuine deterioration in an outcome that was supposed to improve, or if the intervention leads young people to report outcomes differently (for example due to increased awareness of a construct or increased tendency to overpathologise symptoms; Foulkes & Andrews, 2023).

Future studies should examine these possible mechanisms, potentially using a mixed‐method approach, to explain why negative outcomes occur in school mental health interventions (Peters et al., 2023). For example, qualitative methods could be used to investigate how outcomes might relate to specific intervention components (Foulkes & Stapley, 2022), and instrumental variable analysis could be used to investigate whether specific intervention components are causally related to improvements or deterioration in symptoms (Swanson, Hernán, Miller, Robins, & Richardson, 2018). Other experimental or naturalistic procedures assessing exposure to mental health content (e.g simulation experiments in virtual reality or experiments designed around mental health awareness materials) could also be useful to test the hypothesis that increased exposure to mental health information, as would happen in a school‐based mental health intervention, leads to greater reporting of symptoms (Foulkes & Andrews, 2023). Future research should focus on long‐term measurement of negative outcomes to distinguish between mild or transient negative experiences and more severe, enduring harmful outcomes. Furthermore, better measures and reporting guidelines are needed to distinguish between different types of harm.

Overall, this scoping review contributes to a growing number of papers that voice concern or skepticism around the effectiveness of school‐based mental health interventions (Cuijpers, 2022; Dekkers & Luman, 2024; Mansfield et al., 2023), particularly universal interventions (i.e. those delivered to all students and regardless of need). This aligns with findings from the current scoping review: all negative outcomes were found in universal interventions. The current findings add weight to the argument that teaching mental health lessons to all young people might not be useful or appropriate, given that these trials generally show either null effects, negative outcomes or small positive effects, and that the evidence for negative outcomes tends to be overrepresented in different subgroup analyses and in higher‐quality studies. The limited effectiveness of universal mental health interventions could be due to a wide range of factors: young people often do not use the techniques they are taught in interventions because they think they are boring or irrelevant (Montero‐Marin et al., 2023; Peters et al., 2023); the generic information shared in group interventions might be inadequate for those with more severe mental health problems (Dariotis et al., 2016; Foulkes & Stapley, 2022; Hailwood, 2020); interventions do not address the socioeconomic context that may be contributing to young people's mental health problems (Mansfield et al., 2023); and/or school staff do not have the time or resources required to deliver the intervention optimally (Dekkers & Luman, 2024). Yet, despite the evidence of negative outcomes, we must also consider the context of potential benefits and harm‐to‐benefit ratio. It is important to be aware of potential harms when benefits are minimal or non‐existent and to consider whether the benefits truly outweigh any potential harms. Thus, understanding the school context, population needs and individual factors and harm‐to‐benefit ratio should be paramount in informing which interventions should be delivered (if any) in any particular school setting.

There are a number of limitations of the current review that should be noted. First, the review was focused specifically on interventions designed to reduce internalising symptoms or improve wellbeing, and on interventions based on CBT and/or mindfulness. It is therefore unclear whether potential harm (negative outcomes and adverse events) occurs in mental health interventions that are aimed at reducing externalising problems or that use other therapeutic principles or components. There is some indication that this may be an important future line of enquiry: for example, a recent study found that a school intervention based on dialectical behavioural therapy led to an increase in depressive and anxiety symptoms and a deterioration in parent–child relationship quality (Harvey, White, Hunt, & Abbott, 2023). In addition, this review excluded CBT and/or mindfulness interventions that had been adapted to specific populations (e.g. autistic young people, refugees); this is an important direction for future research, especially given that the current review identified several subgroup effects. Since this is a scoping review, we did not conduct a meta‐analysis, and thus cannot comment on the average effect sizes for negative outcomes. Moreover, other outcome statistics that might indicate negative effects, such as reliable deterioration (Cuijpers et al., 2021), are rarely applied in school mental health interventions and could be an avenue for future research. The longevity of negative effects from school mental health interventions also remains unclear, and again this is a key target for future research. Lastly, we used Cochrane's ROB tools to evaluate the quality of studies. While these tools address all the fundamental methodological issues in a randomised controlled trial, they are lacking more precise evaluations for the measurement and reporting of adverse effects and negative outcomes; further studies should explore additional means of evaluating these issues in school trials.

## Conclusions

This scoping review provides the first evaluation of potential harms (negative outcomes and adverse events) from school‐based mental health interventions, focusing specifically on interventions based on CBT and/or mindfulness techniques that aimed to reduce internalising symptoms or improve wellbeing. Overall, our study suggests that a minority (8.93%) of these interventions finds at least one negative outcome and that, to date, no adverse events linked to the intervention itself have been reported. However, publications that were rated high quality (i.e. lower risk of bias) were more likely to report negative outcomes, and a number of studies found that, even when effects were null or positive overall, negative outcomes occurred in subgroups of young people. Future research should examine the mechanisms and intervention components proposed by authors that may be associated with negative outcomes and plan to test whether potential harms are particularly relevant for specific subpopulations.

## Funding information

CGH, RD and LF were funded by a Prudence Trust Research Fellowship awarded to LF (CQR02370). JLA was funded by Wellcome (227640/Z/23/Z).

## Conflict of interest statement

The authors have declared that they have no competing or potential conflicts of interest.

## Ethics statement

The current study used publicly available data and therefore was exempt from ethics approval or written informed consent procedures.

## Trial registration

This scoping review was pre‐registered with the Open Science Framework (OSF; https://osf.io/r9f4q) on 24 July 2023, following PRISMA guidelines for scoping reviews (Tricco et al., 2018). Deviations from our protocol are noted in Supplement S1.

## Supporting information


**Figure S1.** Quality assessment for all studies and those reporting at least one negative effect.
**Table S1.** Query strings by database.
**Table S2.** Inclusion and exclusion of educational settings.
**Table S3.** Combined and adapted modalities excluded from analysis.
**Table S4.** Excluded studies.
**Table S5.** Risk of bias assessment for all studies.
**Table S6.** Characteristics of interventions that found negative outcomes.
**Supplement S1.** Deviations from pre‐registered protocol.
**Supplement S2.** Methods.
**Supplement S3.** Risk of bias assessment.
**Supplement S4.** Details of outcomes for included studies.

## Data Availability

Data sharing not applicable to this article as no datasets were generated or analysed during the current study.
