# Supplementary material for: Scoping review: potential harm from school‐based group mental health interventions
Source: Child Adolesc Ment Health. 2025 Mar 18;30(3):208–22. doi: 10.1111/camh.12760 (PMC12351197; doi:10.1111/camh.12760)
Supplement: Supplementary file 1 — Figure S1. Quality assessment for all studies and those reporting at least one negative effect. Table S1. Query strings by database. Table S2. Inclusion and exclusion of educational settings. Table S3. Combined and adapted modalities excluded from analysis. Table S4. Excluded studies. Table S5. Risk of bias assessment for all studies. Table S6. Characteristics of interventions that found negative outcomes. Supplement S1. Deviations from pre‐registered protocol. Supplement S2. Methods. Supplement S3. Risk of bias assessment. Supplement S4. Details of outcomes for included studies. [file CAMH-30-208-s001.docx]

### **Supporting Information**

**Supplement S1.** Deviations from pre-registered protocol (*p. 2*)

**Supplement S2.** Methods (*pp. 3-16*)

**Table S1**. Query strings by database

**Table S2**. Inclusion and exclusion of educational settings

**Table S3**. Combined and adapted modalities excluded from analysis

**Table S4**. Excluded studies

Additional details on study screening

**Supplement S3**. Risk of bias assessment (*pp. 17-22*)

**Table S5**. Risk of bias assessment for all studies

**Figure S1**. Quality assessment for all studies and those reporting at least one

negative effect

**Supplement S4.** Details of outcomes for included studies (*pp 23-31*)

Additional details of outcomes for included studies

**Table S6.** Characteristics of interventions that found negative outcomes

### **Supplement S1. Deviations from pre-registered protocol**

In the protocol, we stated that we would include studies that ‘include principles based on either mindfulness meditation, cognitive behavioural therapy (CBT) or both’. We later refined this to specify that the interventions must include principles based on mindfulness meditation, CBT or both in their non-adapted form, e.g. these interventions were not adapted to include yoga or adapted for a special needs population (see exclusion criteria and Supplementary Table S3 for more detail). Further deviations included the exclusion of articles not written in English, as this criterion was missed in the protocol, and the use of Kappa rather than Cronbach’s alpha to calculate interrater reliability, which was mislabelled in the protocol. Due to time constraints, the risk of bias assessment strategy was changed. In the protocol, we stated that two authors (CGH and RD) would both conduct the risk of bias assessment on all studies. Instead, two authors (CGH and JLA) conducted 50% of assessments each.

## **Supplement S2. Methods**

## **Table S1.**

## *Query strings by database*

| **Database** | | **Query string** |
| --- | --- | --- |
| ProQuest | Education Collection^a^ | title(intervention* OR program* OR trial OR train* OR curriculum) AND noft(school*) AND noft(mindfulness OR meditation OR cbt OR “cognitive behavio* therapy”) AND noft(mental OR depress* OR mood OR anxi* OR internali?ing OR “emotional problem*” OR “emotional functioning” OR “emotional symptom*” OR” emotional difficult*” OR wellbeing OR well-being) |
|  | Dissertations & Theses Global^b^ |  |
| Ovid | Embase | (intervention* or program* or trial or train* or curriculum).ti. and (school*)ti,ab. and (mindfulness or meditation or cbt or cognitive behavio* therapy)ti.ab. and (mental or depress* or mood or anxi* or internali?ing or emotional problem* or emotional functioning or emotional symptom* or emotional difficult* or wellbeing or well-being).ti,ab. |
|  | Medline |  |
|  | PsycINFO |  |
| EBSCO | ERIC | ( TI intervention* OR TI program* OR TI trial OR TI train* OR TI curriculum ) AND ( TI school* OR AB school* ) AND ( TI mindfulness OR TI meditation OR TI cbt OR TI "cognitive behavio* therapy" OR AB mindfulness OR AB meditation OR AB cbt OR AB "cognitive behavio* therapy" ) AND ( TI mental OR TI depress* OR TI anxi* OR TI mood OR TI internali?ing OR TI “emotional problem*” OR TI “emotional functioning” OR TI “emotional symptom*” OR TI “emotional difficult*” OR TI wellbeing OR TI well-being OR AB mental OR AB depress* OR AB anxi* OR AB mood OR AB internali?ing OR AB “emotional problem*” OR AB “emotional functioning” OR AB “emotional symptom*” OR AB “emotional difficult*” OR AB wellbeing OR AB well-being ) |
| SCOPUS | SCOPUS | title(intervention* OR program* OR trial OR train* OR curriculum) AND title,abst,key(school*) AND title,abst,key (mindfulness OR meditation OR cbt OR "cognitive behavio* therapy") AND title,abst,key (mental OR depress* OR mood OR anxi* OR internali?ing OR "emotional problem*" OR "emotional functioning" OR "emotional symptom*" OR "emotional difficult*" OR wellbeing OR well-being) |
| PsycINFO | Author Search | (seely hayley d or seely h).au. OR (stallard p or stallard paul).au. OR  (kuyken w or kuyken willem).au. OR (andrews j or andrews jack or andrews jack l).au. OR (wigelsworth m or wigelsworth michael).au. |

*Note*: Records known to the researchers were hand-searched in Psyarxiv; All searches were placed limits of 1960 and English Language; ^a^ excluding ERIC collection (as searched later in EBSCO); ^b^ Only including doctoral thesis.

**Table S2.**

## *Inclusion and exclusion of educational settings*

| **Type of educational setting** | **Include (YES/NO)** |
| --- | --- |
| Mainstream school | YES |
| Pre-school | YES |
| Boarding school | YES |
| Orphanage school | YES |
| Transfer school | YES |
| Vocational school | YES |
| Sports academy | YES |
| School-based health centre | YES |
| After-school programme | YES |
| Special educational school | NO |
| Pupil referral unit/alternative education | NO |
| Remote learning | NO |
| Home-schooling | NO |

## **Table S3.**

## *Combined and adapted interventions excluded from analysis*

| **Modality** | **Modifier** | **Category** |
| --- | --- | --- |
| Mindfulness | Combined with | Acceptance and commitment therapy (ACT) |
|  |  | Art |
|  |  | Autogenic training |
|  |  | Dialectical behaviour therapy (DBT) |
|  |  | Health education |
|  |  | Metacognitive training |
|  |  | Narrative therapy |
|  |  | Philosophy |
|  |  | Physical activity |
|  |  | Play |
|  |  | Positive psychology practice |
|  |  | Self-acceptance skills training |
|  |  | Social emotional learning (SEL) |
|  |  | Yoga |
|  | Adapted for | Disordered eating |
|  |  | Mathematics anxiety |
|  |  | Poor sleep/insomnia |
| Cognitive behavioural therapy (CBT) | Combined with | Academic skills training |
|  |  | Art/music therapy |
|  |  | Attention bias modification |
|  |  | Dialectical behaviour therapy (DBT) |
|  |  | Interpersonal psychotherapy |
|  |  | Motivational interviewing |
|  |  | Nutrition and exercise counselling |
|  |  | Pet-assisted therapy |
|  |  | Play therapy |
|  |  | Problem-solving skills training |
|  |  | Sleep hygiene education |
|  |  | Social emotional learning |
|  |  | Social skills training |
|  |  | Yoga |
|  | Adapted for | Anger |
|  |  | Autism |
|  |  | Performance anxiety |
|  |  | Personality types |
|  |  | Poor sleep/insomnia |
|  |  | Social anxiety |
|  |  | Test anxiety |
|  |  | Trauma |
|  |  | Visually impaired children |

**Table S4.**

*Risk of bias assessment for all studies*

| **Year** | **Authors** | **Title** | **Exclusion reason** |
| --- | --- | --- | --- |
| 2023 | Abarkar et al. | The effectiveness of adolescent-oriented mindfulness training on academic burnout and social anxiety symptoms in students: experimental research | Missing information |
| 2014 | Ajaero, Osarenren, and Ilogu | Effectiveness of two intervention strategies on the psychosocial problems of victims of sibling maltreatment among junior secondary students in Lagos, Nigeria | Not CBT or MBT |
| 2013 | Albright et al. | An evaluation of an interdisciplinary rural school mental health programme in Appalachia | One-to-one format |
| 2023 | Albright, Austin, and Mrug | Effects of mindfulness-based intervention on mental health outcomes in youth who are underserved | No control group |
| 2021 | Alqarni and Hammad | Effects of a mindfulness training program on the impulsivity among students with learning disabilities | Irrelevant aim |
| 2011 | Anand and Sharma | Impact of a mindfulness-based stress reduction program on stress and well-being in adolescents: a study at a school setting | No control group |
| 2014 | Anderson et al. | Cost-effectiveness of classroom-based cognitive behaviour therapy in reducing symptoms of depression in adolescents: a trial-based analysis | Irrelevant aim |
| 2023 | Andreu et al. | The effectiveness of a school mindfulness-based intervention on the neural correlates of inhibitory control in children at risk: a randomized control trial | Irrelevant aim |
| 2018 | Antonson, Thorsén, Sundquist, and Sundquist | Upper secondary school students’ compliance with two internet-based self-help programmes: a randomised controlled trial | Not CBT or MBT |
| 2015 | Asbill and Eltiti | Spiritually oriented interventions: comparing mindfulness and centering prayer as interventions for anxiety reduction | Not for CYP |
| 2012 | Attwood et al. | Universal and targeted computerised cognitive behavioural therapy (Think, Feel, Do) for emotional health in schools: results from two exploratory studies. | Not an intervention |
| 2023 | Augustus and Zizzi | Mindfulness in the sport academy classroom: exploring benefits and barriers of a low-dose intervention | Not CBT or MBT |
| 2013 | Barnes and Smith | An examination of the influence of student characteristics on the effectiveness of the Tools For Getting Along curriculum | Irrelevant aim |
| 2014 | Barry, Murphy, and di Blasi | Research into the potential of short interventions in promoting well-being among adolescents | No control group |
| 2016 | Bernay et al. | Pause, breathe, smile: a mixed-methods study of student well-being following participation in an eight-week, locally developed mindfulness program in three New Zealand schools | No control group |
| 2017 | Blake et al. | A cognitive-behavioral and mindfulness-based group sleep intervention improves behavior problems in at-risk adolescents by improving perceived sleep quality | Not in schools |
| 2016 | Blake et al. | The SENSE study: post intervention effects of a randomized controlled trial of a cognitive-behavioral and mindfulness-based group sleep improvement intervention among at-risk adolescents | Not CBT or MBT |
| 2021 | Blouchou | Anxiety disorders in adolescents with dyslexia: incidence and interventions | Embargo/inaccessible |
| 2017 | Bluth, Roberson, and Girdler | Adolescent sex differences in response to a mindfulness intervention: a call for research | Not in schools |
| 2023 | Bookman and Klein | Finding bliss: the development and implementation of a culturally-based mindfulness intervention within an urban Title I elementary school context | Irrelevant aim |
| 2019 | Brown et al. | School-based early intervention for anxiety and depression in older adolescents: a feasibility randomised controlled trial of a self-referral stress management workshop programme | Not CBT or MBT |
| 2018 | Brunwasser, Freres, and Gillham | Youth cognitive-behavioral depression prevention: testing theory in a randomized controlled trial | Irrelevant aim |
| 2022 | Bryning and Edwards | Exploring the economic case for universal and targeted mindfulness-based approaches to prevention: the trial feasibility stage | More relevant paper with sample included |
| 2016 | Burke and Loeber | Mechanisms of behavioral and affective treatment outcomes in a cognitive behavioral intervention for boys | Not in schools |
| 2017 | Burke, Prendeville, and Veale | An evaluation of the “FRIENDS for Life” programme among children presenting with autism spectrum disorder | Not in schools |
| 2017 | Cameron and Gilbert | Effect of a mindfulness intervention on early primary school children with subclinical ADHD | Irrelevant aim |
| 2015 | Campbell and Lanthier | The impact of a school mindfulness program on adolescent stress, wellbeing, and emotion regulation, with attachment as a moderator | More relevant paper with sample included |
| 2017 | Carey | Mindful or mind full? The effectiveness of a small scale mindfulness-based intervention in a mainstream primary school with Year 4 children | No control group |
| 2022 | Caron et al. | Intervention adherence and self-efficacy as predictors of child outcomes in school nurse–delivered interventions for anxiety | One-to-one format |
| 2019 | Carr and Stewart | Effectiveness of school-based health center delivery of a cognitive skills building intervention in young, rural adolescents: potential applications for addiction and mood | No control group |
| 2019 | Carr and Stewart | School-based health center delivery of a cognitive behavioral skills training intervention for anxiety, depression, and executive function in rural adolescents: a feasibility and effectiveness study | No control group |
| 2023 | Carro et al. | Hair cortisol reduction and social integration enhancement after a mindfulness-based intervention in children | Irrelevant aim |
| 2017 | Casson | A mixed methods pilot study evaluating the effectiveness of a mindfulness-based intervention for exam-related anxiety in secondary school settings | No control group |
| 2014 | Challen, Machin, and Gillham | The UK Resilience Programme: a school-based universal nonrandomized pragmatic controlled trial | Not CBT or MBT |
| 2015 | Chan | Children with generalized anxiety disorder: developing a mindfulness intervention | One-to-one format |
| 2018 | Cheng et al. | Do parent mental illness and family living arrangement moderate the effects of the Aussie Optimism program on depression and anxiety in children? | Irrelevant aim |
| 2022 | Chick et al. | A school-based health and mindfulness curriculum improves children’s objectively measured sleep: a prospective observational cohort study | Not CBT or MBT |
| 2012 | Clarke | An evaluation of a brief school-based cognitive behavioural therapy programme for children with ASD | Not CBT or MBT |
| 2023 | Coetzee et al. | Four Steps To My Future (4STMF): acceptability, feasibility and exploratory outcomes of a universal school-based mental health and well-being programme, delivered to young adolescents in South Africa | No control group |
| 2016 | Cotton et al. | Mindfulness-based cognitive therapy for youth with anxiety disorders at risk for bipolar disorder: a pilot trial | Not in schools |
| 2018 | Crowley et al. | Innovations in practice: group mindfulness for adolescent anxiety – results of an open trial | No control group |
| 2021 | Culang et al. | Changing minds: a pilot feasibility study of mindfulness training for at-risk adolescents | Not for CYP |
| 2020 | De Jonge-Heesen et al. | Randomized control trial testing the effectiveness of implemented depression prevention in high-risk adolescents | Not CBT or MBT |
| 2021 | De Jonge-Heesen et al. | Secondary outcomes of implemented depression prevention in adolescents: a randomized controlled trial | Not CBT or MBT |
| 2020 | de la Torre-Luque et al. | Effects of a transdiagnostic cognitive behaviour therapy-based programme on the natural course of anxiety symptoms in adolescence | Not in schools |
| 2017 | Devcich, Rix, Bernay, and Graham | Effectiveness of a mindfulness-based program on school children’s self-reported well-being: a pilot study comparing effects with an emotional literacy program | Active control |
| 2019 | di Blasi et al. | The effectiveness, feasibility and acceptability of a mindfulness-based intervention in two Irish primary schools. | Active control |
| 2017 | Donovan, Spence, and March | Does an online CBT program for anxiety impact upon sleep problems in anxious youth? | Not in schools |
| 2017 | Dove and Costello | Supporting emotional well-being in schools: a pilot study into the efficacy of a mindfulness-based group intervention on anxious and depressive symptoms in children | No control group |
| 2023 | Dunlap, Hart, and Howell | An analysis of the impact of the Inner Explorer mindfulness program on the academic growth of elementary students | Not CBT or MBT |
| 2022 | Dunning et al. | The impact of mindfulness training in early adolescence on affective executive control, and on later mental health during the COVID-19 pandemic: a randomised controlled trial | More relevant paper with sample included |
| 2021 | Egbegi, Bella-Awusah, Omigbodun, and Ani | A controlled trial of cognitive behavioural therapy-based strategies for insomnia among in-school adolescents in southern Nigeria | Not CBT or MBT |
| 2019 | Eiraldi et al. | Pilot study for the fidelity, acceptability, and effectiveness of a PBIS program plus mental health supports in under-resourced urban schools | No control group |
| 2017 | Emerson, Rowse and Sills | Developing a Mindfulness-Based Program for Infant Schools: Feasibility, Acceptability, and Initial Effects | No control group |
| 2014 | Essau et al. | Integrating video-feedback and cognitive preparation, social skills training and behavioural activation in a cognitive behavioural therapy in the treatment of childhood anxiety | Not CBT or MBT |
| 2019 | Essau et al. | Evaluating the real-world effectiveness of a cognitive behavior therapy-based transdiagnostic program for emotional problems in children in a regular school setting | Not CBT or MBT |
| 2019 | Etherington and Costello | Comparing universal and targeted delivery of a mindfulness-based program for anxiety in children | No control group |
| 2019 | Fatania | An initial evaluation of a group school-based therapeutic intervention for low-level symptoms of anxiety in adolescents | No control group |
| 2016 | Fayyad | Resilience-building for children exposed to war trauma: a controlled classroom-based intervention | Not CBT or MBT |
| 2023 | Fernandes et al. | A pilot randomized controlled trial to evaluate a cognitive behavioral videogame intervention: empowered | Irrelevant aim |
| 2023 | Fernandes, Wright, and Essau | The role of emotion regulation and executive functioning in the intervention outcome of children with emotional and behavioural problems | Not CBT or MBT |
| 2020 | Fernández-Martínez et al. | One-year follow-up effects of a cognitive behavior therapy-based transdiagnostic program for emotional problems in young children: a school-based cluster-randomized controlled trial | Not CBT or MBT |
| 2019 | Fernández-Martínez et al. | Effectiveness of the program super skills for life in reducing symptoms of anxiety and depression in young Spanish children | Not CBT or MBT |
| 2013 | Franco, Cangas, Mañas, and Gallego | Exploring the effects of a mindfulness program for students of secondary school | Not CBT or MBT |
| 2020 | Garcí­a Escalera et al. | Educational and wellbeing outcomes of an anxiety and depression prevention program for adolescents | Irrelevant aim |
| 2023 | Ghasemi, Emadian, and Hasanzadeh | Comparison of the effectiveness of mindfulness training and internet-based acceptance and commitment training on academic engagement, mental time travel, and students’ anxiety during the COVID-19 pandemic | Not CBT or MBT |
| 2015 | Gilleece | How can a school based mindfulness intervention help teenage girls who have experienced school related anxiety within the last year? | No control group |
| 2012 | Ginsburg et al. | Treating anxiety disorders in inner city schools: results from a pilot randomized controlled trial comparing CBT and usual care | One-to-one format |
| 2020 | Ginsburg et al. | School-Based Treatment for Anxiety Research Study (STARS): a randomized controlled effectiveness trial | One-to-one format |
| 2013 | Goalen, Bond, and Squires | An exploration of a mindfulness intervention with 7 and 8-year-old children | Irrelevant aim |
| 2020 | Goldingay et al. | Implicit play or explicit cognitive behaviour therapy: the impact of intervention approaches to facilitate social skills development in adolescents | Irrelevant aim |
| 2019 | Goldstein and Quirk | Stop, Collaborate, and Breathe: an examination of the impact of a novel teacher centered classroom-based mindfulness intervention for elementary aged students | Irrelevant aim |
| 2021 | Gómez-Odriozola and Calvete | Effects of a mindfulness-based intervention on adolescents’ depression and self-concept: the moderating role of age | Not in schools |
| 2017 | Gormez et al. | Evaluation of a school-based, teacher-delivered psychological intervention group program for trauma-affected Syrian refugee children in Istanbul, Turkey | No control group |
| 2013 | Green | An evaluation of a FRIENDS for Life programme in a mainstream secondary school and its impact on emotional distress, anxiety and coping skills | Not an intervention |
| 2018 | Hancock et al. | Acceptance and commitment therapy versus cognitive behavior therapy for children with anxiety: outcomes of a randomized controlled trial | Not in schools |
| 2023 | Haugland, Wergeland, and Husabø | School-based cognitive behavioral interventions for youth with anxiety | More relevant paper with sample included |
| 2017 | Haygeman and Clark | An adaptation of the Mindful Schools Curriculum for adolescents: feasibility and preliminary effectiveness on stress, depression, and mindfulness of adolescents in an after-school setting | No control group |
| 2022 | Helminen et al. | Stress-buffering effects of mindfulness programming for adolescents in schools during periods of high- and low-stress | Not CBT or MBT |
| 2019 | Henderson and Norwich | Exploring the impact of a mindfulness-based intervention in relation to primary school children’s mathematics anxiety | Not CBT or MBT |
| 2022 | Henry et al. | The feasibility of a school nurse-led mindfulness program | No control group |
| 2009 | Hudson et al. | Cognitive-behavioral treatment versus an active control for children and adolescents with anxiety disorders: a randomized trial | Not in schools |
| 2023 | Hyseni Duraku et al. | Reducing STEM test anxiety through classroom mindfulness training for lower secondary school children: a pilot study | No control group |
| 2019 | Idsoe, Keles, Olseth, and Ogde | Cognitive behavioral treatment for depressed adolescents: results from a cluster randomized controlled trial of a group course | Not CBT or MBT |
| 2019 | Ishikawa et al. | Developing the universal unified prevention program for diverse disorders for school-aged children | Irrelevant aim |
| 2021 | Ito et al. | The effects of short-term mindfulness-based group intervention utilising a school setting for Japanese adolescents with trauma | No control group |
| 2019 | Janz, Dawe, and Wyllie | Mindfulness-based program embedded within the existing curriculum improves executive functioning and behavior in young children: a waitlist controlled trial. | Irrelevant aim |
| 2020 | Johnstone et al. | Classroom-based mindfulness training reduces anxiety in adolescents: acceptability and effectiveness of a cluster-randomized pilot study | Not CBT or MBT |
| 2014 | Jones et al. | Social anxiety and self-concept in children with epilepsy: a pilot intervention study | Not in schools |
| 2010 | Joyce et al. | Exploring a mindfulness meditation program on the mental health of upper primary children: a pilot study | No control group |
| 2023 | Juul, Bonde, and Fjorback | Altered self-reported resting state mediates the effects of mindfulness-based stress reduction on mental health: a longitudinal path model analysis within a community-based randomized trial with 6-months follow-up | Not for CYP |
| 1990 | Kahn et al. | Comparison of cognitive-behavioral, relaxation, and self-modeling interventions for depression among middle-school students | Not CBT or MBT |
| 2022 | Kambara, Kira, Kohno, and Ogata | Preliminary investigation of the feasibility of a long-term but low-frequency preventive intervention for depression in Japanese high schools | No control group |
| 2023 | Kang and Kim | Development and Evaluation of a Blended Learning Mindfulness Program for High School Students During the COVID-19 Pandemic | Irrelevant aim |
| 2022 | Kaushik and Jena | Effect of cognitive behavioral intervention on electroencephalographic band powers of children with learning difficulty under eyes-closed and eyes-open conditions | Irrelevant aim |
| 2022 | Kay and Young | Distanced from others, connected to self: online mindfulness training fosters psychological well-being by cultivating authenticity | Not for CYP |
| 2021 | Keles and Idsoe | Six- and twelve-month follow-up results of a cluster randomized controlled trial of a CBT-based group course | Not CBT or MBT |
| 2022 | Kennedy et al. | The effect of mindfulness training on resting-state networks in pre-adolescent children with sub-clinical anxiety related attention impairments | Not in schools |
| 2006 | Keogh, Bond, and Flaxman | Improving academic performance and mental health through a stress management intervention: outcomes and mediators of change | Not CBT or MBT |
| 2019 | Kirk et al. | Dose response effects of cognitive-behavioral therapy in a school mental health program | One-to-one format |
| 2012 | Kley, Heinrichs, Bender, and Tuschen-Caffier | Predictors of outcome in a cognitive-behavioral group program for children and adolescents with social anxiety disorder | Not in schools |
| 2022 | Klim-Conforti and Levitt | A school-based cognitive behavioural therapy skills intervention for suicide prevention and wellbeing in youth | More relevant paper with sample included |
| 2020 | Klim-Conforti et al. | The feasibility of a Harry Potter-based cognitive behavioural therapy skills curriculum on suicidality and well-being in middle schoolers | No control group |
| 2021 | Koncz, Köteles, Demetrovics, and Takacs | Benefits of a mindfulness-based intervention upon school entry: a pilot study | Irrelevant aim |
| 2022 | Kuyken et al. | Effectiveness of universal school-based mindfulness training compared with normal school provision on teacher mental health and school climate: results of the MYRIAD cluster randomised controlled trial | Irrelevant aim |
| 2015 | Kösters al. | Indicated prevention of childhood anxiety and depression: results from a practice-based study up to 12 months after intervention | No control group |
| 2020 | Lam and Seiden | Effects of a brief mindfulness curriculum on self-reported executive functioning and emotion regulation in Hong Kong adolescents | Not CBT or MBT |
| 2021 | Laundy, Friberg, Osika, and Chen | Mindfulness-based intervention for children with mental health problems: a 2-year follow-up randomized controlled study | Not in schools |
| 2016 | Lee et al. | School-based interventions for anxious children: long-term follow-up | Not CBT or MBT |
| 2014 | Lillevoll et al. | Uptake and adherence of a self-directed internet-based mental health intervention with tailored e-mail reminders in senior high schools in Norway | One-to-one format |
| 2022 | Lindenberg, Kindt, and Szász-Janocha | Effectiveness of cognitive behavioral therapy-based intervention in preventing gaming disorder and unspecified internet use disorder in adolescents: a cluster randomized clinical trial | Not CBT or MBT |
| 2019 | Lombas et al. | Impact of the Happy Classrooms programme on psychological well-being, school aggression, and classroom climate | Not CBT or MBT |
| 2020 | Lomholt et al. | Feasibility study of Back2School, a modular cognitive behavioral intervention for youth with school attendance problems | One-to-one format |
| 2018 | Lu, Rios, and Huang | Mindfulness, emotion and behaviour: an intervention study with Chinese migrant children | No control group |
| 2017 | Luxford, Hadwin, and Kovshoff | Evaluating the effectiveness of a school-based cognitive behavioural therapy intervention for anxiety in adolescents diagnosed with autism spectrum disorder | Not CBT or MBT |
| 2011 | Makarushka and Murray | Efficacy of an internet-based intervention targeted to adolescents with subthreshold depression | Not in schools |
| 2017 | Malboeuf-Hurtubise et al. | A mindfulness-based intervention pilot feasibility study for elementary school students with severe learning difficulties: effects on internalized and externalized symptoms from an emotional regulation perspective | No control group |
| 2021 | Martínez et al. | Feasibility and acceptability of “Cuida tu Ánimo” (Take Care of Your Mood): an internet-based program for prevention and early intervention of adolescent depression in Chile and Colombia | One-to-one format |
| 2019 | Martinsen et al. | Prevention of anxiety and depression in school children: effectiveness of the transdiagnostic EMOTION program | Not in schools |
| 2010 | Mashmoushi | An evaluation of a brief school-based intervention based on cognitive behaviour therapy to raise pupils’ self-esteem and emotional well-being | Embargo/inaccessible |
| 2016 | Masia Warner et al. | Can school ounsellors deliver cognitive-behavioral treatment for social anxiety effectively? A randomized controlled trial | Not CBT or MBT |
| 2014 | Mazurek Melnyk, Kelly, and Lusk | Outcomes and feasibility of a manualized cognitive-behavioral skills building intervention: group cope for depressed and anxious adolescents in school settings | No control group |
| 2019 | McGovern, Arcoleo, and Melnyk | Cope for asthma: outcomes of a cognitive behavioral intervention for children with asthma and anxiety | No control group |
| 2013 | McNally, Lincoln, Brown, and Chavira | The Coping Cat program for children with anxiety and autism spectrum disorder: a pilot randomized controlled trial | Not in schools |
| 2016 | Mearns and Richards | An exploration of a school-based programme of mindfulness exercises in relation to the social and emotional wellbeing of children | No control group |
| 2021 | Melero et al. | Influence of implementation fidelity on the effectiveness of a t-CBT program targeting emotional problems in childhood | Not CBT or MBT |
| 2020 | Meyer and Eklund | The impact of a mindfulness intervention on elementary classroom climate and student and teacher mindfulness: a pilot study | Irrelevant aim |
| 2013 | Michael et al. | Does cognitive behavioural therapy in the context of a rural school mental health programme have an impact on academic outcomes? | One-to-one format |
| 2021 | Milaré et al. | Mindfulness-based versus story reading intervention in public elementary schools: effects on executive functions and emotional health | Active control |
| 2011 | Miller et al. | Transporting a school-based intervention for social anxiety in Canadian adolescents | Not CBT or MBT |
| 2011 | Miller et al. | An effectiveness study of a culturally enriched school-based CBT anxiety prevention program | Not CBT or MBT |
| 2021 | Mills and Thompson | Supporting disaster impacted youth: evaluation of a universally delivered online mindfulness intervention | Not in schools |
| 2018 | Mio, Matsumuto, et al. | A single-session universal mental health promotion program in Japanese schools: a pilot study | Active control |
| 2021 | Monsillion, Zebdi, and Romo | Implementation and evaluation of a mindfulness-based program for children in a school setting in France | Conference abstract |
| 2023 | Montero-Marin et al. | Do adolescents like school-based mindfulness training? Predictors of mindfulness practice and responsiveness in the MYRIAD Trial. | Irrelevant aim |
| 2013 | Motton | Mindfulness and rumination: mediators of change in depressive symptoms? A preliminary investigation of a universal mindfulness intervention for adolescents | More relevant paper with sample included |
| 2019 | Mrazek et al. | Mindfulness-based attention training: feasibility and preliminary outcomes of a digital course for high school students | No control group |
| 2017 | Muggeo, Stewart, Drake, and Ginsburg | A school nurse-delivered intervention for anxious children: an open trial | No control group |
| 2018 | Murray | Testing two models of delivering and maintaining life skills training in a secondary school setting | No control group |
| 2022 | Nelson et al. | Effects of a regional school-based mindfulness programme on students’ levels of wellbeing and resiliency | No control group |
| 2021 | Nguyen | Dispositional mindfulness and mindfulness training with Vietnamese children : an integrative neurodevelopmental study | Embargo/inaccessible |
| 2015 | O’Callaghan and Cunningham | Can a targeted, group-based CBT intervention reduce depression and anxiety and improve self-concept in primary-age children? | No control group |
| 2021 | Oka et al. | Changes in self-efficacy in Japanese school-age children with and without high autistic traits after the universal unified prevention program: a single-group pilot study | No control group |
| 2007 | O’Kearney, et al | A CBT internet program for depression in adolescents (MoodGym): effects on depressive symptoms, attributional style, self-esteem and beliefs about depression. | Embargo/inaccessible |
| 2020 | Olton-Weber, Hess, and Ritchotte | Reducing levels of perfectionism in gifted and talented youth through a mindfulness intervention | Irrelevant aim |
| 2016 | Ooi et al. | The efficacy of a group cognitive behavioral therapy for war-affected young migrants living in Australia: a cluster randomized controlled trial | Not CBT or MBT |
| 2020 | Orgiles et al. | How does a CBT-based transdiagnostic program for separation anxiety symptoms work in children?: effects of Super Skills for Life | No control group |
| 2023 | Orgilés et al. | Effectiveness of a transdiagnostic computerized self-applied program targeting children with emotional problems: a randomized controlled trial | Not in schools |
| 2020 | Orgilés et al. | Effectiveness of video-feedback with cognitive preparation in improving social performance and anxiety through Super Skills for Life programme implemented in a school setting | Not CBT or MBT |
| 2022 | Palmer and Kober | A randomized controlled trial investigating dose-response and moderating relationships of changes in psychological wellbeing in single-session mindfulness meditation interventions | Not in schools |
| 2023 | Portele and Jansen | The effects of a mindfulness-based training in an elementary school in Germany | Irrelevant aim |
| 2018 | Pössel, Smith, and Alexander | LARS&LISA: a universal school-based cognitive-behavioral program to prevent adolescent depression | Not an intervention |
| 2015 | Pucci and Przeworski | The Girls Link program: an examination of the efficacy of a brief prevention program for behaviorally inhibited female adolescents | Not in schools |
| 2021 | Putwain and von der Embse | Cognitive-behavioral intervention for test anxiety in adolescent students: do benefits extend to school-related wellbeing and clinical anxiety | Not CBT or MBT |
| 2014 | Rawlett and Thomas | Effect of a nurse-led mindfulness intervention with at-risk adolescents | Irrelevant aim |
| 2021 | Razza, Bergen-Cico, Reid, and Uveges | The benefits of mindfulness for promoting resilience among at-risk adolescents: results from the Inner Strength Teen Program | Irrelevant aim |
| 2023 | Reaven et al. | Implementing school-based cognitive behavior therapy for anxiety in students with autism or suspected autism via a train-the-trainer approach: results from a clustered randomized trial | Not CBT or MBT |
| 2019 | Redfern et al. | Innovations in practice: CUES-Ed: an in-service evaluation of a new universal cognitive behavioural early mental health intervention programme for primary school children | Not CBT or MBT |
| 2020 | Redziniak and Block-Lerner | Using curriculum-based mindfulness workshops to foster openness to diversity and challenge | Irrelevant aim |
| 2023 | Reichart and Bohanon | Measuring the effect of an intervention on student engagement and reading comprehension | Irrelevant aim |
| 2015 | Robinson et al. | The safety and acceptability of delivering an online intervention to secondary students at risk of suicide: findings from a pilot study | No control group |
| 2023 | Rowe et al. | Short research article: changes in life functioning in a self-help, online program for child and adolescent anxiety | No control group |
| 2017 | Rush et al. | The effects of a mindfulness and biofeedback program on the on- and off-task behaviors of students with emotional behavioral disorders | Not CBT or MBT |
| 2018 | Salum et al. | Group cognitive behavioral therapy and attention bias modification for childhood anxiety disorders: a factorial randomized trial of efficacy | Not in schools |
| 2018 | Sanders et al. | An implementation of a computerized cognitive behavioral treatment program to address student mental health needs: a pilot study in an after-school program | One-to-one format |
| 2016 | Sanger | A neurophysiological investigation of mindfulness training in secondary schools: modifications in cognitive control and emotion processing in adolescents | More relevant paper with sample included |
| 2016 | Sanger and Dorjee | Mindfulness training with adolescents enhances metacognition and the inhibition of irrelevant stimuli: evidence from event-related brain potentials | Irrelevant aim |
| 2022 | Scaini et al. | The Cool Kids as a School-Based Universal Prevention and Early Intervention Program for Anxiety: Results of a Pilot Study | No control group |
| 2010 | Schonert-Reichl and Lawlor | The effects of a mindfulness-based education program on pre- and early adolescents’ well-being and social and emotional competence | Not CBT or MBT |
| 2023 | Shanok et al. | Mindfulness-training in preadolescents in school: the role of emotionality, EEG in theta/beta bands, creativity and attention | No control group |
| 2017 | Shapiro, Heath, and Talwar | Effectiveness of stressoff strategies: a single-session school-based stress management program for adolescents | No control group |
| 2022 | She et al. | Planting seeds for resilience – a pilot mindfulness program in migrant Chinese children | Conference abstract |
| 2012 | Sibinga et al. | A randomized, active-controlled trial of school-based mindfulness instruction for urban middle-school male youth | Conference abstract |
| 2015 | Silverstone et al. | Initial findings from a novel school-based program, EMPATHY, which may help reduce depression and suicidality in youth | Not CBT or MBT |
| 2017 | Silverstone et al. | Long-term results from the Empowering a Multimodal Pathway Toward Healthy Youth program, a multimodal school-based approach, show marked reductions in suicidality, depression, and anxiety in 6,227 students in grades 6-12 (aged 11-18) | Not CBT or MBT |
| 2016 | Skryabina et al. | Effect of a universal anxiety prevention programme (FRIENDS) on children’s academic performance: results from a randomised controlled trial. | Irrelevant aim |
| 2023 | Smarinsky, Brown, and Christian | Examining the effects of a mindfulness-based intervention using a neurofeedback device on adolescent introspection: a quasi-experimental time-series design | Irrelevant aim |
| 2015 | Smith et al. | Computerised CBT for depressed adolescents: randomised controlled trial | One-to-one format |
| 2012 | Sohn and Lee | Effects of a cognitive behavioral therapy program on mental health problems in children dealing with trauma: focused on community district victimized by oil spill | Not in English |
| 2011 | Stallard et al. | Computerized CBT (Think, Feel, Do) for depression and anxiety in children and adolescents: outcomes and feedback from a pilot randomized controlled trial | Not in schools |
| 2005 | Stallard et al. | An evaluation of the FRIENDS programme: a cognitive behaviour therapy intervention to promote emotional resilience | No control group |
| 2007 | Stallard et al. | The FRIENDS emotional health programme: initial findings from a school-based project | No control group |
| 2008 | Stallard et al. | The FRIENDS emotional health prevention programme: 12-month follow-up of a universal UK school-based trial | No control group |
| 2012 | Stallard et al. | Classroom based cognitive behavioural therapy in reducing symptoms of depression in high risk adolescents: pragmatic cluster randomised controlled trial | More relevant paper with sample included |
| 2014 | Stasiak et al. | A pilot double blind randomized placebo controlled trial of a prototype computer-based cognitive behavioural therapy program for adolescents with symptoms of depression | One-to-one format |
| 2023 | Stewart and Bernard | Empowering the victims of bullying: the Bullying: The Power to Cope program | Not CBT or MBT |
| 2013 | Storch et al. | The effect of cognitive-behavioral therapy versus treatment as usual for anxiety in children with autism spectrum disorders: a randomized controlled trial | Not in schools |
| 2009 | Sykes | An evaluation of the FRIENDS program: a therapeutic intervention for anxious young people | Embargo/inaccessible |
| 2020 | Tenaglia and DiGiuseppe | Effectiveness and mechanisms of change of mindfulness and relaxation training delivered in a high school | Not CBT or MBT |
| 2011 | Tillfors et al. | A randomized trial of internet-delivered treatment for social anxiety disorder in high school students | Not in schools |
| 2016 | Tomyn, Fuller-Tyszkiewicz, Richardson, and Colla | A comprehensive evaluation of a universal school-based depression prevention program for adolescents | Not CBT or MBT |
| 2023 | Tornivuori et al. | Accessible mental well-being intervention for adolescents in school settings: a single-group intervention study using a pretest-posttest design | No control group |
| 2023 | Tran et al. | School-based universal mental health promotion intervention for adolescents in Vietnam: two-arm, parallel, controlled trial | Not CBT or MBT |
| 2020 | Tran et al. | School-based, two-arm, parallel, controlled trial of a culturally adapted resilience intervention to improve adolescent mental health in Vietnam: study protocol | Not an intervention |
| 2023 | Underwood et al. | Identifying and changing cognitive vulnerability in the classroom: preliminary evaluation of CUES-Ed, a school-based universal cognitive behavioural early intervention service for 7 – 10 year olds | Irrelevant aim |
| 2016 | Urao et al. | Effectiveness of a cognitive behavioural therapy-based anxiety prevention programme for children: a preliminary quasi-experimental study in Japan | Not in schools |
| 2020 | Van Der Mheen et al. | Cognitive behavioural therapy for anxiety disorders in young children: a Dutch open trial of the Fun FRIENDS program | No control group |
| 2013 | van Starrenburg et al. | Effectiveness and underlying mechanisms of a group-based cognitive behavioural therapy-based indicative prevention program for children with elevated anxiety levels | Not an intervention |
| 2021 | Visagie, Loxton, Swartz, and Stallard | Cognitive behaviour therapy-based early intervention and prevention programme for anxiety in South African children with visual impairments | Not CBT or MBT |
| 2019 | Vliek, Overbeek, and de Castro | Effects of Topper Training on psychosocial problems, self-esteem, and peer victimisation in Dutch children: a randomised trial | Not in schools |
| 2017 | Whittaker | Evaluating the impact of a mindfulness based intervention on Year 5 children’s social and emotional skills, optimism and mindful awareness | No control group |
| 2012 | Whittaker et al. | MEMO – a mobile phone depression prevention intervention for adolescents: development process and postprogram findings on acceptability from a randomized controlled trial | Not in schools |
| 2022 | Winters and Barwa | The behavioral and academic achievement impacts of mindfulness interventions for urban elementary school students | Irrelevant aim |
| 2019 | Wright, Roberts, and Proeve | Mindfulness-based cognitive therapy for children (MBCT-C) for prevention of internalizing difficulties: a small randomized controlled trial with Australian primary school children | Active control |
| 2013 | Yabko and Tracey | Examining the efficacy of the Ninja Mind Training program (NMT): a mindfulness-based intervention for bullied teens | Irrelevant aim |
| 2016 | Yeo, Goh, and Liem | School-based intervention for test anxiety | Not CBT or MBT |
| 2021 | Young et al. | Personalized depression prevention: a randomized controlled trial to optimize effects through risk-informed personalization | Not in schools |

**Additional details on Study screening**

From 118 records assessed for negative events, 1 record (De Voy, 2019) reports 3 separate studies, 1 record (Miller et al., 2011) reports 2 separate studies, and 8 records (Ahlen et al., 2018; Bernstein et al., 2005; Britton et al., 2015; Klim-Conforti et al., 2021; Kuyken et al., 2022; Lowry-Webster et al., 2001; Sportel et al., 2013; Volanen et al., 2020) report duplicate interventions (Ahlen et al., 2019; Berstein et al., 2008; Kang et al., 2018; Klim-Conforti et al., 2023; Montero-Marin et al., 2022; Lowry-Webster et al., 2003; de Hullu et al., 2017; Saarinen et al., 2022) respectively. These include records reporting on additional follow up timepoints or subgroup analysis from the main studies.

**Supplement S3. Risk of bias assessment**

**Table S5.**

*Risk of bias assessment for all studies*

| Study ID (author) | Bias arising from randomisation process | Bias arising from recruitment | Bias arising from deviations to intervention | Bias arising due to missing outcome data | Bias arising due to measurement of the outcome | Bias arising due to selection of reported result | Overall risk of bias |
| --- | --- | --- | --- | --- | --- | --- | --- |
| Ahlen 2018 | Low | Low | Low | Low | Low | Low | Low |
| Ahlen 2019 | Some concerns | Some concerns | Low | Some concerns | Some concerns | Low | High |
| Alampay | Some concerns | Low | N/A | Some concerns | Some concerns | Some concerns | High |
| Amin | Low | Low | N/A | Low | Low | Some concerns | Some concerns |
| Andrews | Low | Low | Low | Low | Low | Low | Low |
| Are | Some concerns | Low | Low | Low | Some concerns | Low | High |
| Aydin | Some concerns | Some concerns | N/A | Low | Some concerns | Some concerns | High |
| Batra sabey | Some concerns | Low | N/A | Some concerns | Some concerns | Some concerns | High |
| Bernstein 2005 | Low | Low | Low | Low | Some concerns | Low | Some concerns |
| Bernstein 2008 | Low | Low | Low | Low | Some concerns | Low | Some concerns |
| Bogaert | Some concerns | Low | Low | Low | Some concerns | Low | Some concerns |
| Britton | Low | Low | Low | Low | Some concerns | Low | Some concerns |
| Broderick | High | High | N/A | Low | High | Some concerns | High |
| Calear | Some concerns | Low | Low | Low | Low | Low | Some concerns |
| Campbell | Some concerns | Low | Low | Some concerns | Some concerns | Low | High |
| De hullu | Low | Low | Low | Low | Some concerns | Low | Some concerns |
| De voy (study 1) | Some concerns | Low | Low | Low | Some concerns | Low | High |
| De voy (study 2) | Some concerns | Low | Low | Low | Some concerns | Low | High |
| De voy (study 3) | Some concerns | Low | Low | Low | Some concerns | Low | High |
| Dobson | Some concerns | Some concerns | N/A | Low | Some concerns | Some concerns | High |
| Duffy | High | High | Low | Some concerns | Some concerns | Low | High |
| Fite | High | Low | N/A | Low | Some concerns | Low | High |
| Folch | Some concerns | Low | Low | Low | Some concerns | Some concerns | High |
| Frank | Low | Low | Low | Low | Low | Low | Low |
| Fung 2019 | Low | Low | Low | Low | Some concerns | Low | Some concerns |
| Fung 2016 | Some concerns | Low | N/A | Low | Some concerns |  | High |
| Gaete | Low | Low | N/A | Low | Some concerns | Low | Some concerns |
| Gallegos | Some concerns | Low | Low | Low | Some concerns | Some concerns | High |
| Garcia-rubio | Some concerns | Low | Low | Low | Some concerns | Low | High |
| Garmy | High | Some concerns | Low | Low | Some concerns | Low | High |
| Ghiroldi | High | Low | Some concerns | Low | High | Low | High |
| Gregor | Some concerns | Low | N/A | Some concerns | Some concerns | Low | High |
| Haugland | Low | Low | N/A | Low | Some concerns | Low | Some concerns |
| Horowitz | Some concerns | Low | N/A | Low | High | Some concerns | High |
| Huppert | Some concerns | Low | Low | Low | Some concerns | Low | High |
| Johnson 2016 | Low | Low | Low | Low | Some concerns | Low | Some concerns |
| Johnson 2017 | Low | Low | Low | Low | Some concerns | Low | Some concerns |
| Johnson 2019 | Some concerns | Low | Low | Some concerns | Some concerns | Low | High |
| Johnson 2021 | Low | Low | Low | Low | Some concerns | Low | Some concerns |
| Kaltwasser | Some concerns | Some concerns | Low | Some concerns | Some concerns | Low | High |
| Kang | Low | Low | Low | Low | Some concerns | Low | Some concerns |
| Kato | Some concerns | Low | Low | Low | Some concerns | Low | High |
| Kennes | Some concerns | Low | Low | High | Low | Some concerns | High |
| Khalid | Low | Low | Low | Some concerns | Some concerns | Low | High |
| Khan | Some concerns | Low | N/A | High | High | Some concerns | High |
| Klim-conforti (2021) | Low | Low | Some concerns | Low | Some concerns | Low | High |
| Klim-conforti (2023) | Low | Low | Some concerns | Low | Some concerns | Low | High |
| Kul | Some concerns | Low | N/A | High | High | Some concerns | High |
| Kuyken (2013) | Some concerns | Low | Low | Low | Low | Low | Some concerns |
| Kuyken (2022) | Low | Low | Low | Low | Low | Low | Low |
| Lake | High | Low | N/A | Low | High | Some concerns | High |
| Lam | Low | Low | N/A | Low | High | High | High |
| Lau | Some concerns | High | N/A | High | High | Some concerns | High |
| Liu | Low | Low | Low | Low | Some concerns | Low | Some concerns |
| Lowe | Low | Low | Low | High | Some concerns | Low | High |
| Lowry-webster (2001) | Some concerns | Low | Low | Low | Low | Low | Some concerns |
| Lowy-webster (2003) | Some concerns | Some concerns | Low | Some concerns | Low | Low | High |
| Lu | Low | Low | N/A | Low | High | Some concerns | High |
| Mackenzie | Some concerns | Low | Low | Low | Some concerns | Low | High |
| Martinsen | Low | Low | Low | High | Low | Low | High |
| Matsumoto | Some concerns | Low | Low | Low | Low | Low | Some concerns |
| Miller | Some concerns | Low | Low | Some concerns | Some concerns | Some concerns | High |
| Miller | Some concerns | Low | Low | Some concerns | Some concerns | Some concerns | High |
| Modi | Low | High | N/A | Low | Some concerns | Some concerns | High |
| Montero-marin | Low | Low | Low | Low | Low | Low | Low |
| Mostafazadeh | High | Low | Low | High | Some concerns | Some concerns | High |
| Muris | Some concerns | Low | N/A | Low | Some concerns | Some concerns | High |
| Nobel | Low | Low | Low | Low | Low | Low | Low |
| O’connor | Low | Low | Low | Low | Low | Low | Low |
| O’kearney | Some concerns | Low | Low | Low | Some concerns | Low | High |
| Ohira | High | Low | Low | Low | High | Low | High |
| Pahl | Low | Low | Low | Some concerns | Low | Low | Some concerns |
| Paul | Some concerns | Low | Some concerns | Low | Some concerns | Low | High |
| Perry | Low | Low | Low | Low | Low | Low | Low |
| Poli | Low | Low | N/A | Low | Low | Low | Low |
| Pophillat | Low | Low | Low | Low | Some concerns | Low | Some concerns |
| Poppelaars | Low | Low | Low | Low | High | Low | High |
| Possel | Low | Low | Low | Low | Some concerns | Some concerns | High |
| Potek | Low | Some concerns | N/A | High | High | Some concerns | High |
| Quach | Some concerns | Low | N/A | Low | Low | Low | Some concerns |
| Raes | High | Low | Low | Low | High | Low | High |
| Reiss | Some concerns | Low | N/A | Low | Some concerns | Some concerns | High |
| Reynolds | Some concerns | Low | N/A | Some concerns | High | Some concerns | High |
| Ricarte | Some concerns | Some concerns | N/A | High | High | Some concerns | High |
| Rice | Some concerns | Low | Low | Low | Some concerns | Low | High |
| Rice | Some concerns | Some concerns | N/A | High | High | Some concerns | High |
| Rodgers | Low | Low | Low | Some concerns | Some concerns | Low | High |
| Ruocco | Some concerns | Low | Low | Low | Low | Low | Some concerns |
| Saarinen | Some concerns | Low | Low | Some concerns | Some concerns | Some concerns | High |
| Sanger | Some concerns | Low | Low | Low | Low | Low | Some concerns |
| Saw | Low | Low | Low | Low | Some concerns | Low | Some concerns |
| Saw | Some concerns | Some concerns | N/A | Low | High | Some concerns | High |
| Scarfuto 2022 | Low | Low | Low | Some concerns | Some concerns | Low | High |
| Scarfuto 2023 | Low | Low | Low | Low | Some concerns | Low | Some concerns |
| Seeley | Low | Low | Low | Low | Some concerns | Low | Some concerns |
| Shochet | Some concerns | Some concerns | Low | High | Low | High | High |
| Sibinga | Low | Low | Low | Some concerns | Some concerns | Low | High |
| Simpson | Some concerns | Low | N/A | High | High | Some concerns | High |
| Sinyor | Some concerns | High | Some concerns | Low | Some concerns | Low | High |
| Spence | Low | Low | Low | Low | Low | Low | Low |
| Sportel | Low | Low | Low | Low | Low | Low | Low |
| Stallard 2012 | Low | Low | Low | Low | Low | Low | Low |
| Stallard 2014 | Low | Low | Low | Low | Low | Low | Low |
| Stoppelbein | Low | Low | Low | Some concerns | Some concerns | Low | High |
| Theurel | Low | Low | Low | Low | Some concerns | Low | Some concerns |
| Thompson | Some concerns | Some concerns | N/A | Low | High | Some concerns | High |
| Tien | Low | Low | Low | Some concerns | Some concerns | Low | High |
| Urao 2018 | High risk | Low | Low | Low | Low | Low | High |
| Urao 2021 | Some concerns | Low | High | Low | Some concerns | Low | High |
| Urao 2022 | Some concerns | Low | Low | Low | Some concerns | Low | High |
| Van de weijer-bersma | Low | Low | Low | Low | Some concerns | Low | Some concerns |
| Van starrenburg | Low | Low | Low | Low | Some concerns | Low | Some concerns |
| Vickery | Some concerns | Low | Low | Low | Some concerns | Low | High |
| Volanen | Low | Low | Low | Low | Low | Low | Low |
| Waters | Low | Low | Low | Low | Some concerns | Some concerns | High |
| Wigelsworth | Low | Low | Low | Low | Some concerns | Low | Some concerns |
| Wijnhoven | Low | Low | Low | Low | Low | Low | Low |
| Willenbrink | Some concerns | Low | Low | Low | Some concerns | Low | High |
| Wong | Low | Low | Low | High | Low | High | High |
| Zandi | Some concerns | Low | N/A | Low | High | Some concerns | High |

**Figure S1.**

*Quality assessment for total studies (n = 120) and those reporting at least one negative effect (n = 11)*

*Note*: For reference, lower risk = higher quality.

**Supplement S4. Details of outcomes for included studies**

***Andrews et al., 2022: Universal CBT-based intervention***

This study described a cluster-randomised controlled trial of a universal CBT-based intervention for adolescents aged 12-14. At baseline the sample consisted of 3,200 participants. This study found negative effects in the primary outcome, such that CBT training led to an increase in internalising symptoms at the 6-month and 12-month timepoints but had no effects at the 18-month timepoint (two negative and one null effect). This study also found that CBT was not more effective than the control at reducing any of the secondary outcomes (8 null effects total) and had mixed effects on other secondary outcomes that were not pre-registered for analysis (four null effects, four positive effects total).

***Frank et al., 2021: Universal mindfulness-based intervention***

This study describes a cluster-randomised controlled trial of a mindfulness intervention for adolescents aged 15-16. At baseline the sample consisted of 251 students. This study found negative effects in one secondary outcome such that the intervention decreased participants’ goal directed behaviour (one negative effect) at post-intervention compared to the control group. This study also found that the intervention generally had no effect on other primary and secondary measures compared to the control (28 null effects, 1 positive effect). Neurocognitive computer-based tasks are not reported here.

***Johnson, Burke, Brinkman and Wade, 2016: Universal mindfulness-based intervention***

This study describes a cluster-randomised controlled trial of a universal mindfulness intervention for adolescents aged 13-14. At baseline the sample consisted of 308 students. This study found negative effects in subgroup analysis, such that males, those with low levels of depression and those with weight/shape concerns experienced increased anxiety in the intervention relative to the equivalent individuals in the control group at the 3-month follow-up (three negative effects). The study also found that the intervention had no effects compared to the control on any of the study’s primary outcomes (14 null effects) and other subgroup analysis (23 null effects). No positive effects were reported in this study.

***Johnson and Wade, 2021: Universal mindfulness-based intervention***

This study describes a cluster-randomised controlled trial of a mindfulness intervention for adolescents aged 13-16. At baseline the sample consisted of 434 students. This study found negative effects in the primary outcome at the 3-month follow up, such that participants in the intervention reported worse scores in one of eight facets of mindfulness (decentering and nonreactivity) compared to the control group (one negative effect). There were no differences between the mindfulness and control groups on other outcome measures (23 null effects). This study also found negative effects in subgroup analyses assessing specific age groups. At the 3-month follow up, 13-14 year olds (compared to 15-16 year olds) who received the mindfulness intervention were worse in two aspects of mindfulness (reduced awareness of external environment, reduced decentering and non-reactivity), and in wellbeing compared to controls (three negative effects). The intervention had no effect compared to the control on all other subgroup analysis for age (21 null effects). This study also found negative effects in a subsample of 161 students that participated in an additional 9-month follow up, such that the intervention group had worse scores on one aspect of mindfulness (i.e. they reported lower levels of decentering and nonreactivity) and worse wellbeing at the 3-month follow-up (two negative effects), and worse scores on wellbeing and weight/shape concerns at the 9-month follow-up (two negative effects). There were no other differences between the intervention and control groups on other subsample outcomes (12 null effects). No positive effects were recorded in this study.

***Klim-Conforti et al., 2021: Universal CBT-based intervention***

This study describes a cluster-randomised controlled trial of a CBT intervention for adolescents aged 11-14. At baseline the sample consisted of 603 individuals. The study found negative effects on gender subgroup analysis, such that males in the intervention group had higher impulsivity scores than males in the control group at post-intervention (one negative effect). This study’s main analysis found that the CBT skills intervention was more effective compared to the control at reducing composite suicidality (primary outcome; one positive effect) and most of the secondary outcomes (seven positive effects; two null effects). Other subgroup analysis on gender favoured females compared to males on most outcomes (seven positive outcomes – of which six favour females, six null outcomes).

***Kuyken et al., 2022 & Montero-Marin et al., 2022: Universal mindfulness-based intervention***

These studies describe a cluster-randomised controlled trial of a universal mindfulness intervention for adolescents aged 9-13, in both the main analysis (Kuyken et al., 2022) and the subgroup analysis (Montero-Marin et al., 2022). At baseline the sample consisted of 8,376 participants. In the main study, the authors found negative effects in secondary outcomes such that participants reported an increase in hyperactivity/inattention difficulties at postintervention and 1-year follow-up; an increase in panic disorder symptoms, obsessive-compulsive disorder symptoms and total anxiety at post-intervention; a decrease in mindfulness skills at postintervention; and an increase in teacher-reported emotional symptoms at the 1-year follow up (seven negative effects). The study found that the mindfulness training intervention had no effect on primary outcomes (3 null effects) and other secondary outcomes (52 null effects). The secondary paper also reported negative effects on a subgroup analysis for participants at high risk and at low risk of mental health problems on the three primary outcomes. They found an increase in symptoms of depression and a decrease in wellbeing at post-intervention and at the 1-year follow-up for the high-risk group (four negative effects). There were no differences between conditions for any of the other primary subgroup (eight null effects) or secondary subgroup (12 null effects in total) outcomes. No positive effects were reported across primary and secondary outcomes or subgroup analyses.

***Seely, Gaskins, Pössel and Hautzinger, 2023: Universal CBT-based intervention***

This study describes a cluster-randomised controlled trial of a universal CBT prevention programme for adolescents aged 13-14. At baseline the sample consisted of 646 participants. This study found negative effects on primary outcomes such that the CBT prevention programme was associated with less prosocial behaviour relative to the control condition (one negative effect). The study found that the intervention had mostly no effect compared to the control on other primary outcomes (one positive effect, four null effects).

***Stallard et al., 2012: Universal CBT-based intervention***

This study describes a cluster-randomised controlled trial of a universally delivered CBT intervention for adolescents aged 12-16, aimed at students with high risk of depression (i.e. those with high levels of depressive symptoms). At baseline the sample consisted of 1064 participants identified as being at high risk of depression. This study found negative effects in primary outcome after accounting for variables that were imbalanced at baseline, such that the CBT intervention increased depressive symptoms at the 12-month follow-up compared to teaching-as-usual (one negative effect). The study also found a negative effect in secondary outcomes at 12-months, such that the CBT group had more thoughts of personal failure than the teaching-as-usual control group. This study found that the CBT intervention was not more effective than any of the controls for most secondary outcomes (two positive effects, 27 null effects) and subgroup analysis on the primary outcome (10 null effects).

***Stoppelbein, 2003: Universal CBT-based intervention***

This study, presented in a doctoral thesis, describes a cluster-randomised controlled trial of a universal CBT intervention for 14-15 year olds. At baseline the sample consisted of 127 participants. The study found negative effects on secondary outcomes for a subsample of participants with baseline subclinical depressive scores (i.e. higher levels of depression), such that participants in the CBT intervention reported an increase in dysfunctional attitudes (one negative effect) compared to participants in the control. This study found that the CBT intervention had no effect compared to the control on any of the primary outcomes for the full and subsample of participants (four null effects), secondary outcomes for the full sample (six null effects) and for the subgroup sample (five null effects). No positive effects were reported in this study.

***Wigelsworth et al., 2018: Universal CBT-based intervention***

This study describes a cluster-randomised controlled trial of a universal CBT intervention for young people aged 8-11. At baseline the sample consisted of 3284 participants. This study found a negative effect in the subgroup analysis, such that children eligible for free school meals in the CBT intervention group reported higher scores of depression and anxiety after the intervention than children in the teaching as usual group (one negative effect). This study also found that the CBT intervention had no effect compared to the control on any of the primary outcomes (one null effects), secondary outcomes (three null effects) and other subgroup analysis (seven null effects). No positive effects were reported in this study.

**Table S6.**

*Characteristics of interventions that found negative outcomes*

| **Authors** | **Name** | **Why**  **(rationale/ theory)** | **What (materials and procedures)** | **Who provided** | **How** | **Where** | **When & how much** | **How well (planned/enacted)** |
| --- | --- | --- | --- | --- | --- | --- | --- | --- |
| Andrews et al. 2022 | Climate Schools | Uses CBT to reduce anxiety and depression via skill acquisition, psychoeducation and symptom management (p.5043) | Class lessons, online self-paced activities in school, homework and reinforcement activities  (see Table S1) | Self-completed and  teacher-led (teachers were provided with online and hard-copy manuals containing the activities, lesson syllabus, and implementation guidelines (p.5044) | Universal, online and face-to-face, delivered in groups (p.5043) | Australia | 6 sessions  40 minutes | Teachers completed a fidelity logbook; completion rates for six lessons ranged from 88-97% (Supplement - Fidelity) |
| Frank et al. 2021 | Learning to Breathe | Teach mindfulness- based stress reduction to strengthen emotion regulation and executive functioning (p.1236) | Psychoeducation, class activities (discussions, specific mindfulness-based practices, experiential activities) and homework practices (p.1237 & Figure 1) | Teacher-led (teachers were given practice sessions and a manual with point-by-point listing of activities per lesson) in addition to coaching sessions that included weekly calls and a classroom visit (p.1238). Teachers completed a 6h individual training session and a 2-day training led by programme developers (p.1238) | Universal, face-to-face in groups, at-home practice  (p.1238) | USA | 12 sessions  NA minutes | Independent coders were randomly assigned to code a lesson for fidelity; across sessions and teachers, fidelity to the program was 78% (p.1238) |
| Johnson, Burke, Brinkman, and Wade 2016 | .b | Teach mindfulness to improve anxiety, depression, wellbeing, eating disorder risk and improve transdiagnostic prevention potential (p.2) | Lessons, guided and unguided practices, interactive and experiential teaching, and guided practices for home (see p.3) | Lessons were taught by the first author (using a guided manual) who is a mindfulness practitioner with 10 years of personal practice, and .b certification (p.3) | Universal, face-to-face, in groups and self-guided, at home practice (p.3) | Australia | 8 sessions  35-60 minutes | Adherence and engagement with homework practice was measured via self-report questionnaires; 87% attended at least six of the eight lessons (p.6) |
| Johnson and Wade 2021 | Mindfulness Training for Teens | Teach mindfulness to  improve anxiety, depression, eating disorder risk and wellbeing (p. 2474) | Interactive introductions to mindfulness concepts, informal mindfulness practices, guided  meditations and facilitator-guided group discussion (p.2476) | Mindfulness lessons were taught by the first author who is a mindfulness practitioner with 15 years of relevant practice, and .b certification (p.2476) | Universal, face-to-face, in groups (p.2476) | Australia | 8 sessions  65-75 minutes | Fidelity was assessed during the pilot study and involved other authors using a marking rubric (MBI-TAC). Randomly selected audio recordings of lessons were also reviewed by the programme developer; average lesson rating was “proficient” and ranged from 4.7-5.5/6 in the pilot (p.2476 and Supplement) |
| Klim-Conforti et al. 2021 | Harry Potter CBT Skills Curriculum | Use CBT to reduce suicidality by emphasizing resilience and practical skill acquisition since portrayals of mastery and coping in the media can help reduce suicidal behaviours and deaths (p.135) | Intervention follows a protocolized manual based on reading the third book in the Harry Potter series (p.135) | Intervention was delivered by teachers who were trained for half a day on CBT and modules using the book (p.136) | Universal, face-to-face, in groups (p.136) | Canada | NA sessions  NA minutes | Lead investigator visited each class to assess fidelity which was deemed to be adequate. No formal protocol for monitoring fidelity was implemented (p. 136) |
| Kuyken et al 2022  (subgroup analysis: Montero-Marin et al. 2022) | .b | Teach mindfulness skills that support young people’s resilience (p.101) | Curriculum with psychoeducation, class discussions, and mindfulness practices. Supplemented with worksheets and online mindfulness practices and at-home practice (pg.101) | Intervention was delivered by school teachers who received training and practice, and received a course booklet (see Supplement A) | Universal, face-to-face, in groups, and at home-practice (p.102) | UK | 10 sessions  30-50 minutes | All classes were recorded, and a randomly selected subset were rated by independent assessors using the MBI-TAC (pg.102, Supplement B); on average, teachers were rated as delivering the intervention competently and adhered to 83% of the standardised curriculum (p.104) |
| Seely, Gaskins, Pössel, and Hautzinger 2023 | LARS&LISA | The curriculum is based on the social information processing (SIP) model and applies multiple elements of CBT with the aim of preventing depression and improving other peripheral outcomes (p.927) | The programme is embedded in the curriculum and uses, lessons, activities, role-play and videos to demonstrate how Lars & Lisa (characters) use coping skills in difficult situations (p.927) | The prevention program condition was led by a MHP trained in CBT principles (master-level psychologists with a mean of 6.3 years of experience) in one condition and by a non-MHP (teachers with a mean of 11 years of experience) in another condition. Both MHP and teachers completed a 2-day training (p.926). | Universal, face-to-face, in groups (p.927) | Germany | 10 sessions  90 minutes | Each group session was videotaped and reviewed by a licensed clinical psychologist in biweekly, 1.5-hour supervision sessions with group leaders (p.926). Note: fidelity not reported in this study. |
| Stallard et al. 2012 | Resourceful Adolescent Programme UK | Teach CBT to develop skills such as emotion-regulation capacities, coping mechanisms, and thinking styles, which are reported to protect against the development of depression (p.2) | The programme consists of modules and booster sessions (p.2) | Two trained facilitators (graduate students) led each session working alongside the class teacher. The facilitators had appropriate professional backgrounds or experience of working with children or young people (p.2) | Universal, face-to-face, in groups (p.2) | UK | 9 sessions  50-60 minutes | Fidelity was assessed by independent observation of 5% of sessions. Facilitators rated the content of each session on a scale (p.2). Of the 36 sessions observed, 31 covered 100% of the core tasks and 5 covered at least 75% of tasks (p.3) |
| Stoppelbein 2003 | Coping with Depression | Teach CBT to help control mood and promote adaptive coping skills for ongoing life events and stressors (p.16) | A course with lessons, exercises and practice of relaxation techniques (p.16) | The intervention was guided by a leader trained in the principles of cognitive behavioural techniques (p.16) | Universal , face-to-face, in groups (p.16) | USA | 10 sessions  50 minutes | Note: Assessment of fidelity was not reported in this study. |
| Wigelsworth et al. 2018 | FRIENDS for Life | Teach CBT to promote emotional resilience and prevent (or stabilise) the development of negative feelings of anxiety and depression (p.6) | The programme consists of lessons, practice activities and homework activities. The course leader follows a detailed manual whilst pupils are given a workbook (p.7) | The programme was delivered by an external team, The Salus Group (Salus; p.7). Salus officers received 1 day of training and have experience working with young people (p.8) | Universal, face-to-face, in groups, and at home-practice (p.7) | UK | 10 sessions  60-90 minutes | implementation was directly recorded by trained researchers (10 observations, carried out in 10 separate classes (p.18); The overall quality of delivery was rated as high, with a total average score of 8.1/10 (p.39). |

*Note*: following the TIDieR framework, we have not included item 9 ‘Tailoring’ and item 10 ‘Modifications’ since we explicitly excluded studies that were adapted/modified/tailored to address a specific concern. CBT=cognitive-behavioural therapy; MBI-TAC=Mindfulness Based Interventions – Teacher Assessment Criteria; MHP=mental health professional; SBMT=school-based mindfulness training.
